# Complex genetic and histopathological study of 15 patient-derived xenografts of aggressive lymphomas

**DOI:** 10.1038/s41374-022-00784-w

**Published:** 2022-04-29

**Authors:** Radek Jakša, Jana Karolová, Michael Svatoň, Dmitry Kazantsev, Martina Grajciarová, Eva Pokorná, Zbyněk Tonar, Magdalena Klánová, Lucie Winkowska, Diana Maláriková, Petra Vočková, Kristina Forsterová, Nicol Renešová, Alexandra Dolníková, Kristýna Nožičková, Pavel Dundr, Eva Froňková, Marek Trněný, Pavel Klener

**Affiliations:** 1 Institute of Pathology, University General Hospital Prague and First Faculty of Medicine, Charles University, Prague, Czech Republic; 2 Institute of Pathological Physiology, First Faculty of Medicine, Charles University, Prague, Czech Republic; 3 First Department of Medicine- Hematology, University General Hospital Prague and First Faculty of Medicine, Charles University, Prague, Czech Republic; 4 CLIP- Childhood Leukaemia Investigation Prague, Department of Pediatric Haematology and Oncology, Second Faculty of Medicine, Charles University and University Hospital Motol, Prague, Czech Republic; 5 Department of Histology and Embryology and Biomedical Center, Faculty of Medicine in Pilsen, Charles University, Pilsen, Czech Republic

**Keywords:** B-cell lymphoma, Animal biotechnology, Cancer genetics

## Abstract

Non-Hodgkin lymphomas (NHL) represent the most common hematologic malignancies. Patient-derived xenografts (PDXs) are used for various aspects of translational research including preclinical in vivo validation of experimental treatment approaches. While it was repeatedly demonstrated that PDXs keep majority of somatic mutations with the primary lymphoma samples, from which they were derived, the composition of PDX tumor microenvironment (TME) has not been extensively studied. We carried out a comparative genetic and histopathological study of 15 PDX models derived from patients with various types of NHL including diffuse large B-cell lymphoma (DLBCL; *n* = 7), Burkitt lymphoma (BL; *n* = 1), mantle cell lymphoma (MCL; *n* = 2), and peripheral T-cell lymphomas (PTCL; *n* = 5). Whole exome sequencing (WES) of the PDXs and primary lymphoma cells was implemented in 13 out of 15 cases with available DNA samples. Standard immunohistochemistry (IHC) was used to analyze the composition of PDX TME. WES data confirmed that PDXs maintained the genetic heterogeneity with the original primary lymphoma cells. In contrast, IHC analysis revealed the following recurrently observed alterations in the composition of PDX tumors: more blastoid lymphoma cell morphology, increased proliferation rate, lack of non-malignant cellular components including T cells and (human or murine) macrophages, and significantly lower intratumoral microvessel density and microvessel area composed of murine vessels. In addition, PDX tumors derived from T-NHL displayed additional differences compared to the primary lymphoma samples including markedly lower desmoplasia (i.e., the extent of both reticular and collagen fibrosis), loss of expression of cytotoxic granules (i.e., perforin, TIA, granzyme B), or loss of expression of T-cell specific antigens (i.e., CD3, CD4, CD8). Our data suggest that despite keeping the same genetic profiles, PDX models of aggressive NHL do not recapitulate the microenvironmental heterogeneity of the original lymphomas. These findings have implications on the relevance of PDX models in the context of preclinical research.

## Abstract (web summary)

The authors carried out a comparative genetic and histopathological study of 15 patient-derived xenografts (PDX) that were newly established from patients with various types of aggressive non-Hodgkin lymphomas. We have demonstrated that despite keeping the same genetic profiles the PDX models do not recapitulate microenvironmental heterogeneity of the original lymphomas. These findings have implications on the relevance of PDX models in the context of preclinical research.

## Introduction

Non-Hodgkin lymphomas (NHL) represent the most common hematologic malignancies^1,2^. Murine patient-derived xenograft (PDX) models of human malignancies represent relevant tools for various aspects of preclinical and translational research in (hemato)oncology including the study of lymphoma biology, prediction of personalized treatment, or exploration of mechanisms of drug resistance^3,4^. It has been repeatedly demonstrated that PDXs share genetic mutational landscapes with the primary lymphoma cells, from which they were derived^5,6^. In contrast, the composition of the PDX TME and its relevance compared to the original primary TME has been less extensively investigated^7^. Several studies have reported that PDXs maintain the same histopathologic and immunophenotypic features compared to the original lymphoma cells. However, they have largely focused on the expression of lymphoma surface antigens (CD20), disease markers (PAX5, cyclin D1) and proliferation rate by Ki-67^8^. Pathogenesis of cancer, survival of malignant cells, as well as mode-of-activity (MoA) of many types of anti-cancer therapies, however, can be both directly and indirectly impacted by non-malignant cells of the TME (e.g., T-lymphocytes, macrophages, natural killer (NK) cells), or the extent of neovascularization and desmoplasia^9,10^. MoA of T-cell engaging therapies, immunomodulatory agents, therapeutic monoclonal antibodies, antiangiogenic drugs, or liposome-encapsulated cytostatics all depend not only on the biology of the lymphoma cells, but also on the TME factors^11^.

In this study we analyzed 15 newly derived PDX models of NHL and provide their complex genetic and histopathological analysis compared to their respective primary lymphoma tumors (i.e., infiltrated lymph nodes, or extranodal lymphoma masses). Besides whole exome sequencing (WES)-based analysis of landscapes of somatic mutations and predicted copy number variants (CNVs), we provide a comprehensive IHC-based study of the composition of the PDX TME that address not only the PDX lymphoma cells, but also the non-malignant TME components.

## Materials and methods

### Patients and samples

Informed consent to donation of lymphoma samples for the derivation of murine PDXs was obtained from all patients according to the Declaration of Helsinki. The study was approved by the institutional Ethics Committee (48/18). For the purpose of whole exome sequencing, B-NHL and T-NHL samples were sorted for CD19-positive B-cells using CD19 microBeads (Miltenyi Biotec), and CD45-positive cells using CD45 microBeads (Miltenyi Biotec), respectively. Lymphoma cell infiltration of each sample was verified by flow cytometry.

### Establishment of PDXs

The study was approved by the Animal Care and Use Committee (MSMT-21527/2017-8). Female immunodeficient NOD.Cg-*Prkdc*^*scid*^ *Il2rg*^*tm1Wjl*^/SzJ mice (purchased from the Jackson Laboratory, Bar Harbor, Maine, USA, commonly referred to as NSG mice) were maintained in individually ventilated cages at the Center of Experimental Biomodels of the First Faculty of Medicine, Charles University, Prague, Czech Republic. The donated primary lymphoma specimens were cut into small pieces and homogenized through 45-microM nylon mesh, suspended in phosphate buffered saline (PBS), and subcutaneously injected (10–30 × 10^6^ cells/ mouse) into the left abdominal flank of adult female NSG mice. Alternatively, small pieces of lymphoma (approx. 2 × 2 × 2 mm) and/or homogenized lymphoma cells suspended in BD Matrigel Matrix (BD Biosciences) were surgically inserted into sub-renal capsules of mice under anesthesia. When tumors of the SC-injected mice reached 2 cm in the largest dimension or when engraftment of tumors in the sub-renal region became discernible by ultrasound examination (Vevo 3100/LAZR-X), the animals were euthanized, and tumors excised and used for subsequent analyses.

### Next generation exome sequencing and copy number variant analyses

Genomic DNA was extracted using DNeasy Blood & Tissue Kit (QIAGEN, Germany) according to the manufacturer’s protocol. Samples were sequenced by our facility (CLIP, Prague, Czech Republic) on the NextSeq 500 instrument (Illumina, San Diego, CA) according to manufacturer’s protocols with sequencing libraries prepared using the SureSelectXT Human All Exon V6 + UTR kit (Agilent Technologies, Santa Clara, CA). Primary lymphoma samples and PDX samples were analyzed as described previously^12^. Only nonsynonymous variants in the gene coding regions with coverage of at least 10 reads with mapping quality and base quality higher than 20 in all related samples were compared together based on their frequency. Variants present in patient’s germline DNA at frequency higher than 0.05 were excluded from analysis in all cases. We compared variants with an allele fraction ≥0.1 in at least one of the compared samples that were present in at least 3 reads in both the primary sample and the corresponding PDX sample. All variant filtering was done in RStudio and frequencies and counts of variants were plotted using the ggplot2 library. These variants were then manually reviewed in Integrative Genomics Viewer (http://www.broadinstitute.org/igv) to clear sequencing artefacts or variants present but not called in the germline sample. Curated gene lists for each lymphoma subtype among our PDX models (diffuse large B-cell lymphoma, DLBCL, mantle cell lymphoma, MCL, peripheral T-cell lymphomas, PTCL, Burkitt lymphoma, BL) were created based on recent publications of frequently and recurrently mutated genes in the respective diseases and variants present in these genes were selected and marked in resulting diagrams and tables^13–42^. These lists are provided in the Supplementary Table 4 (sheet S1D).

Copy number variants (CNVs) were predicted using CNVkit and a pooled reference from normal control samples^43,44^. Variants with copy numbers higher or lower than 2 were plotted for each patient and PDX sample using the circlize package for R^45^. To generate color-coded tables, CNVs were filtered by a list of genes of interest for CNV (Supplementary Table 4, sheet S1E) and the most prominent changes were plotted.

#### Immunohistochemical analysis of patient lymphoma biopsies and their respective PDX models

The immunohistochemical (IHC) analysis was performed using 4 μm thick sections of formalin-fixed and paraffine-embedded (FFPE) tissue blocks (involved lymph nodes or infiltrated extranodal tissue) obtained from patients with newly diagnosed or treatment-refractory lymphomas, as well as from PDX tissue blocks obtained from lymphoma-bearing mice. The expression of the following antigens was examined: CD45, Ki-67, MYC, human CD31, mouse CD31, human CD68, mouse CD68, CD3, CD4, CD8, CD56, CD20, bcl2, bcl6, CD10, IRF4/MUM1, cyclin D1, SOX11, CD5, ALK, CD23, PD-1, CD7, granzyme B, perforin, TIA-1. The clones, manufacturers, dilution, and staining instruments for all antibodies are summarized in Supplementary Table 1. The immunohistochemical results were assessed semi-quantitatively according to the overall percentage of positive cells (0–100%). For Ki67 scoring, any brown staining of the nucleus above the background was regarded as positive. Global assessment of the whole tumor tissue area was performed. The scoring was based on estimation of areas with high, medium and low Ki-67 index. From each area, at least 100 nuclei were counted. The global score was then calculated for each sample and the results were reported in 10% cut-offs. The presence of Epstein-Barr virus (EBV) infected cells was detected by INFORM EBER (Epstein-Barr virus early RNA) probe on a BenchMark ULTRA automated platform followed manufacturer´s protocol (Ventana Benchmark, Roche, Basel, Switzerland). Reticular and collagen fibers on the tumor background were visualized by Gordon-Sweet and van Gieson staining. Grading of fibrosis was assessed semi-quantitatively as G0 (no fibrosis – scattered fine reticular fibers), G1 (mild fibrosis – loose network of intersecting reticular fibers), G2 (severe fibrosis – dense network of intersecting thick reticular and collagen fibers), or G3 (sclerosis – compact areas of hyalinized collagen).

### Quantification of microvessel density (MVD) and microvessel area (MVA)

Four pairs of tissue blocks were evaluated. In each pair, one tissue block represented a patient’s bioptic sample, the other one represented the corresponding PDX tumor. Five randomly oriented sections from each tissue block were stained immunohistochemically using human and murine CD31 antibodies binding to endothelium of the microvessels (Supplementary Table 1). The histological quantification of microvessels was done as described previously and with sampling optimized according to Kolinko et al.^46,47^. Briefly, 10 fields of view were captured in a systematic uniform way from each section using a 40× objective (Olympus Optical Co., Ltd., Tokyo, Japan) (Supplementary Fig. 1)^48,49^. The intratumoral microvessels were evaluated using MVD and MVA (Supplementary Table 2). MVD of CD31-positive microvessel profiles was estimated using the unbiased CountingFrame^49,50^. MVA was evaluated using a stereological PointGrid and the Cavalieri principle (Supplementary Fig. 2)^50,51^.

These two quantitative parameters are considered as complementary: MVD gives the 2-D density of microvessel profiles per sectional area of the tumor, while the MVA provides information on the fraction occupied by microvessel profiles within the tumor. The histological quantification was done on 4 randomly selected PDX models (VFN-D1, VFN-D5, VFN-M5R1, VFN-R4) and the respective primary lymphoma samples (P3, P5, P9 and P15) using the Ellipse software (ViDiTo, Košice, the Slovak Republic) (Supplementary Table 3).

## Results

### Establishment of PDX tumors

In total, 15 PDXs were derived from 15 patients (9 males, 6 females) with diverse types of NHL at diagnosis (*n* = 5) or at disease relapse (*n* = 10). The PDXs included DLBCL (*n* = 7) including one transformed DLBCL (tDLBCL) from marginal zone lymphoma, and one double-hit lymphoma (with *MYC* and *BCL2* gene rearrangements), BL (*n* = 1), MCL (*n* = 2), angioimmunoblastic T-cell lymphoma (AITL, *n* = 2), peripheral T-cell lymphoma, not otherwise specified (PTCL, NOS, *n* = 1), anaplastic large cell lymphoma (ALCL), anaplastic lymphoma kinase (ALK)-positive (*n* = 1), and ALCL, ALK-negative (*n* = 1). Baseline characteristics of the lymphoma subtypes, disease stage and previous therapies (where applicable) are summarized in Table 1.

**Table 1:** Baseline characteristics of the analyzed samples.

Pt	Gender	Dg	Subtype	Disease status	Sample origin	WES	Therapy	PDX
1	F	DLBCL	non-GC	Dg	LN	Yes	Untreated	VFN-D3
2	M	DLBCL	non-GC	Dg	LN	Yes	Untreated	VFN-D6
3	M	DLBCL	non-GC	R/R	LN	Yes	G-CHOP	VFN-D1
4	M	DLBCL	GC	R/R	EN (soft tissues)	Yes	R-CHOP + venetoclax; R-ESHAP; R-GIFOX; RT	VFN-D4
5	F	DLBCL	non-GC	R/R	LN	Yes	R-CHOP; R-ESHAP; R-GIFOX; RT	VFN-D5
6	M	tDLBCL	GC, transformed from MZL	Dg	LN	No	R-COP	VFN-D12
7	F	DLBCL	double-hit	R/R	LN	Yes	R-CHOP / R-ESHAP	VFN-D20
8	M	Burkitt		R/R	EN (stomach)	Yes	R-Hyper-CVAD, R-MTX-HD-araC	VFN-B3
9	M	MCL		R/R	LN	Yes	Nordic protocol	VFN-M5R1
10	F	MCL		R/R	LN	Yes	Nordic protocol	VFN-M1
11	M	AITL		R/R	LN	Yes	CHOEP	VFN-T3
12	F	AITL		R/R	LN	No	Untreated	VFN-T7
13	F	PTCL, NOS		R/R	LN	Yes	CHOEP	VFN-T6
14	M	ALCL	ALK-negative	Dg	LN	Yes	Untreated	VFN-T5
15	M	ALCL	ALK-positive	Dg	LN	Yes	Untreated	VFN-T4

*AITL* angioimmunoblastic T-cell lymphoma, *ALCL* anaplastic large cell lymphoma, *ALK* anaplastic lymphoma kinase, *araC* cytarabine, *COP* cyclophosphamide; vincristine; prednisone, *CHOP* COP + doxorubicin, *CHOEP* CHOP + etoposide, *Dg* diagnosis, *DLBCL* diffuse large B-cell lymphoma, *EN* extra-nodal, *ESHAP* etoposide; solumedrole; high-dose araC; cisplatin, *FL* follicular lymphoma, *G-CHOP* obinutuzumab plus CHOP, *GC* germinal center, *GIFOX* gemcitabine; ifosfamide; oxaliplatin, *HD-araC* high dose araC, *hyperCVAD* hyperfractionated cyclophosphamide; doxorubicin, vincristine; dexamethasone, *LN* lymph node, *MCL* mantle cell lymphoma, *MZL* marginal zone lymphoma, *MTX* methotrexate, *Pt* patient, *PTCL* peripheral T-cell lymphoma, *R* rituximab, *RM* rituximab maintenance, *R/R* relapsed, *RT* radiotherapy, *tDLBCL* transformed DLBCL.

### PDXs retained most somatic mutations and copy number variants found in the respective primary lymphoma biopsies

WES was implemented in 13 out of 15 cases with available DNA. Non-malignant patients´ DNA was used to filter out gene polymorphisms. WES data confirmed that the established PDX models kept majority of somatic mutations of the original lymphoma cells, from which they were derived (“shared” mutations, Figs. 1, 2, Supplementary Figs. 4–17). A list of all relevant variants detected by WES is shown in Supplementary Table 4 (10.5281/zenodo.6035345). Median allele frequencies of the shared mutations were comparable between the PDXs and the respective primary lymphoma samples (Supplementary Table 5).

**Fig. 1:**
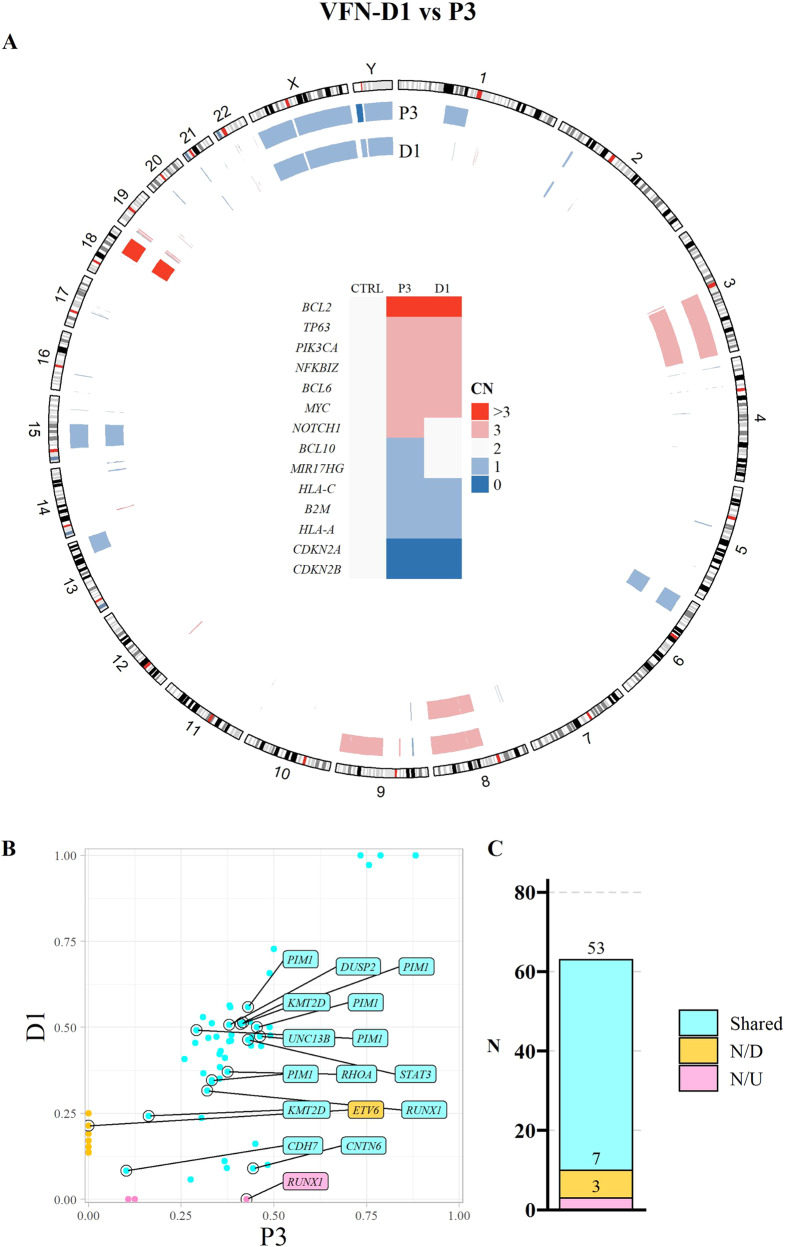
Whole exome sequencing of the original lymphoma cells obtained from patient P1 and the derived PDX model VFN-D3. **A** A circular ideogram showing the predicted copy number variants (CNVs) for the patient’s sample (P1) and the corresponding PDX model (D3). Outer track represents chromosomal positions of the predicted CNVs. Gene deletions are marked in shades of blue (“1”: predicted monoallelic deletion, “0”: predicted biallelic deletion). Gene amplifications are marked in shades of red (“3”: gain of 1 allele, “>3”: gain of more than 1 allele). Graphical table at the center is showing CNV of genes of special interest. **B** Scatter plot showing the allele frequency of shared, newly-detected (N/D), and newly-undetected (N/U) variants in the PDX model sample compared to the sample from which it was derived. Labels show variants found in genes of special interest for the analyzed lymphoma subtype, described in the methods. **C** Stacked bar plot showing numbers of shared, N/D and N/U variants in the patient and the PDX sample. P patient sample, CN copy number, CTRL germline control DNA from patient, N number.

**Fig. 2:**
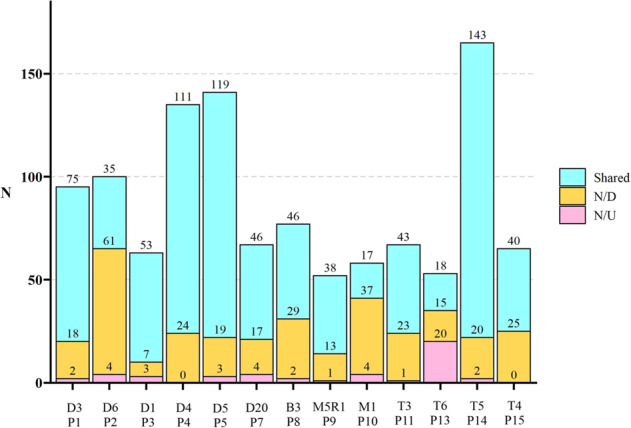
Number of somatic mutations shared, newly detected (N/D), and newly undetected (N/U) in the PDXs compared to the primary lymphoma samples according to WES analysis. A stacked bar plot showing numbers of shared, N/D and N/U variants in all analyzed PDX models (VFN-D3, D6, D1, D4, D5, B3, M5R1, M1, T3, T6, T5, and T4) compared to the primary patient (P) samples.

Somatic mutations that were not detected in the PDX cells but that were present in the primary lymphoma samples (labeled as “newly undetected” [N/U] in PDX, Fig. 2) comprised predominantly low-frequency mutations (median allele frequency 0.15 in primary lymphoma samples, Table 2). These N/U mutations rarely comprised genes from the positive gene list (Supplementary Table 2C).

**Table 2:** Median allele frequencies and types of somatic mutations newly detected or undetected in PDX models.

A.	Median AF of N/D mutations in PDXs	Median AF of N/U mutations in patient samples
All PDX models	0.18	0.15
DLBCL	0.27	0.14
MCL	0.18	0.36
T-NHL	0.15	0.14
BL^a^	0.17	0.11
**B**.	**N/D from the Gene-list**	**N/U from the Gene-list**
VFN-D1 vs P3	*ETV6*	*RUNX1*
VFN-D3 vs P1	*PDGFRA*	0
VFN-D4 vs P4	*NF1, HIPK3*	0
VFN-D5 vs P5	*ITPKB, ENAM*	0
VFN-D6 vs P2	*IRF4, RNF213, MYC, FOXO1, KLHL14, KLHL14, OSBPL10*	0
VFN-D20 vs P7	0	0
VFN-M1 vs P10	0	0
VFN-M5R1 vs P9	0	0
VFN-T3 vs P11	*STAT1, ZNF708, RHOA*	0
VFN-T4 vs P15	*SPEN, FSIP2, DCC, ZNF532*	0
VFN-T5 vs P14	*AIRD1B, VPS13A, MYD88*	0
VFN-T6 vs P13	*IRF2BP2, VAV1, TCF20*	*BIRC6*
VFN-B3 vs P8	0	0

*A* Median allele frequencies (AF) of newly detected (N/D) mutations in PDX models and newly undetected (N/U) mutations in primary lymphoma samples, *B* N/D and N/U variants from the list of genes of special interest (Gene-list), *0* no gene list variant, *BL* Burkitt lymphoma, *DLBCL* diffuse large B-cell lymphoma, *MCL* mantle cell lymphoma, *P1-P15* patient primary lymphoma samples, *T-NHL* T-cell non-Hodgkin lymphomas, *VFN* PDX models derived from primary lymphoma samples P1-P15.

^a^Only 1 PDX model.

Somatic mutations that were detected in the PDX cells but not in the primary lymphoma samples (labeled as “newly detected” [N/D] in PDX, Fig. 2) were observed more frequently than the N/U mutations, but also comprised predominantly low-frequency mutations (median allele frequency 0.18 in the PDXs, Table 2). The N/D mutations often included genes from the positive gene lists (Supplementary Table 2C). The majority of N/D mutations were not detectable in the corresponding primary lymphoma samples (i.e., their allele frequency in the primary lymphoma samples were 0), and mostly consisted of nucleotide transitions (Supplementary Figs. 18 and 19). Some N/D mutations were detected in the primary lymphoma samples, but their allele frequency was below the pre-defined detection threshold (i.e., >0, but <10%).

### Consistently observed phenotypic differences between the primary lymphoma biopsies and the respective PDXs

**Fig. 3:**
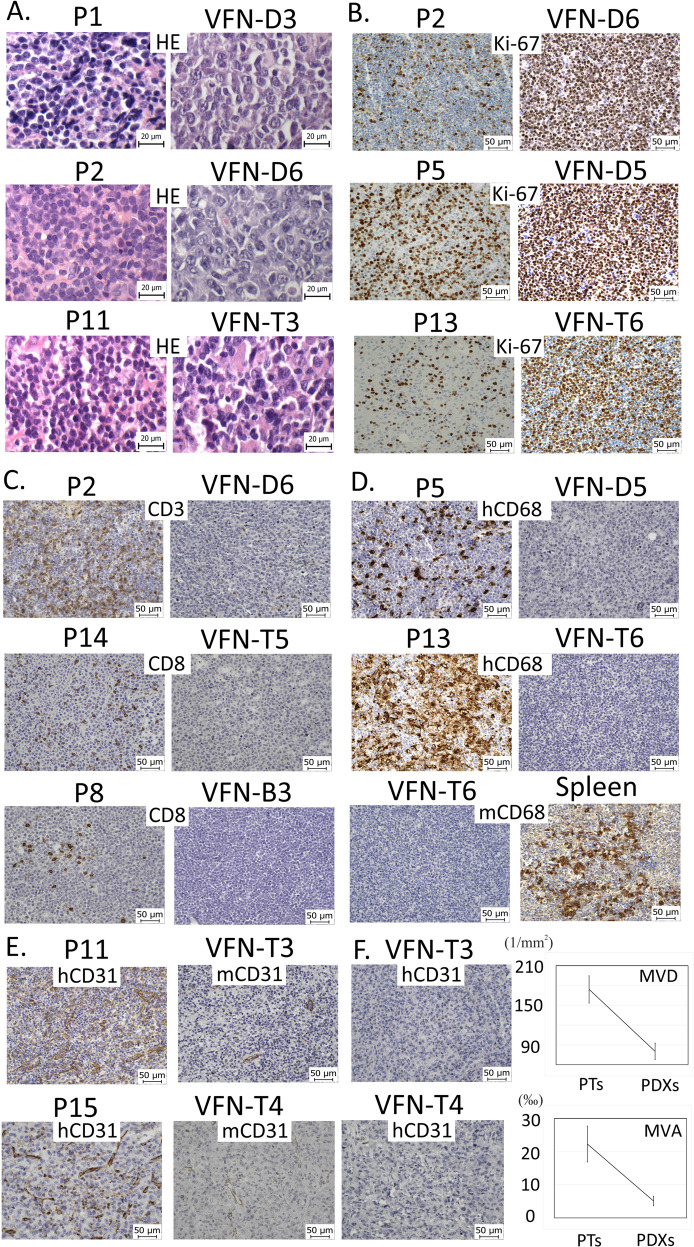
Consistently observed phenotypic alterations between primary lymphoma biopsies and the derived PDX tumors. **A** More blastoid morphology of PDX cells compared to patients´ primary lymphoma cells; **B** Increased proliferation rate by Ki-67 in PDX cells compared to patients´ original lymphoma cells; **C** Lack of T-lymphocytes in the PDX TME; **D** Lack of both human and murine macrophages in PDX TME; presence of murine macrophages in murine spleen; **E** Lack of vessels of human origin; presence of vessels of murine origin in the PDX TME; **F** Significantly lower microvessel density (MVD) and microvessel area (MVA) in PDX tumors compared to original patients´ lymph node biopsies; *Y* axis in MVD displays number of vascular profiles per 1 mm^2^ of the tumor; *Y* axis in MVA displays microvessel area as area fraction (per mill, ‰) of the total area of CD31-positive microvessel profiles within the tumor; the data are displayed as means, bars show the standard deviations.“. For more detailed information, see also Supplementary Tables 2 and 3.

To investigate the composition of the TME, all PDX tumors were analyzed by standard IHC methods and compared to their respective primary samples (Supplementary Table 1, Supplementary Figs. 4–17). Several PDXs were characterized by more aggressive (blastoid) morphology and accelerated proliferation rate measured via Ki-67 expression (Fig. 3A, B). Absence of T-lymphocytes and macrophages was observed in all analyzed PDX tumors (Fig. 3C, D). Moreover, no murine macrophages were detected in the PDX tumors despite their presence in murine spleen, liver, and bone marrow (Fig. 3D). The vasculature of the PDX tumors was composed of vessels of murine origin, and both MVA and MVD were significantly decreased compared to the primary lymph node biopsies (Fig. 3E, F).

### Phenotypic differences between PDX tumors of T-NHL and the primary lymphoma biopsies

**Fig. 4:**
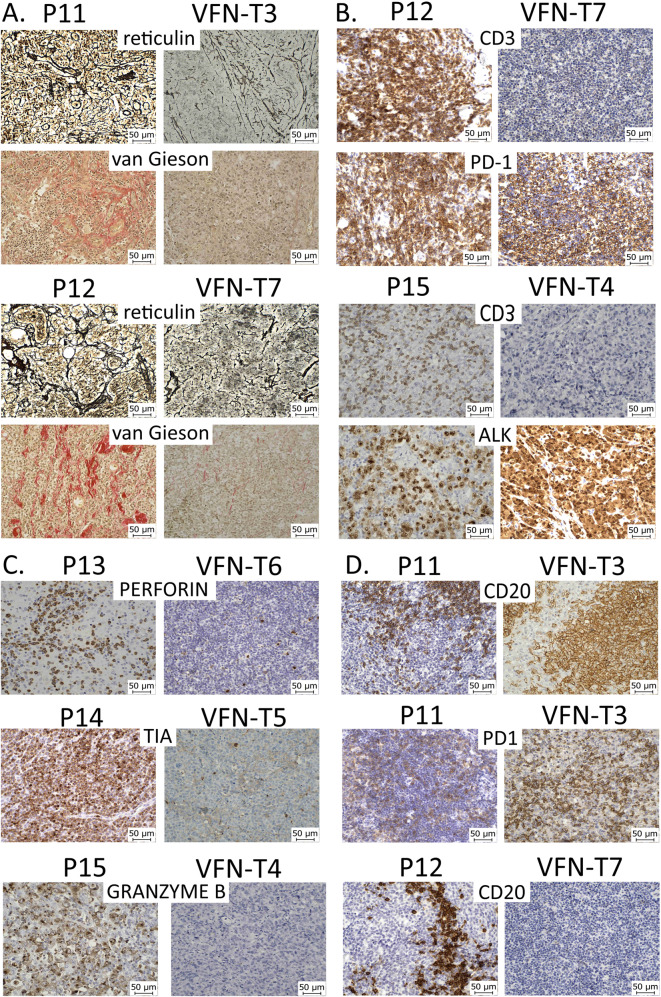
Phenotypic differences between PDX tumors of T-NHL and original lymphoma biopsies. **A** Lower extent of desmoplasia in PDX tumors VFN-T3 and VFN-T7 compared to their respective lymphoma samples, P11 and P12; **B** Loss of expression of the CD3 T-cell marker, but maintained expression of PD-1 and ALK in PDX cells VFN-T7 and VFN-T4 compared to primary T-NHL cells P12 and P15 respectively; **C** Loss of expression of cytotoxic granules (perforin, T-cell intracytoplasmic antigen [TIA], granzyme B) in PDX cells VFN-T6, VFN-T5, and VFN-T4 compared to primary T-NHL cells P13, P14, and P15 respectively; **D** Aberrant expression of CD20 in VFN-T3 PDX cells; absence of CD20-positive B-cells in VFN-T7 PDX tumor compared to the primary lymphoma sample P12.

Significantly lower extent of desmoplasia (reticular and collagen fibrosis) was found in the majority of T-NHL and several B-NHL models (Fig. 4A, Supplementary Figs. 3–17). Additionally, loss of expression of CD3 and other T-cell-specific markers (CD4, CD8), as well as loss of expression of cytotoxic granules (perforin, T-cell-restricted intracellular antigen [TIA], granzyme B) was observed in PDX cells derived from T-NHL (Fig. 4B, C, Supplementary Figs. 15–17). The PDX tumors derived from the two patients with AITL had discrepant expression of CD20 (Fig. 4D). While VFN-T7 had no detectable CD20-positive cells compared to the significant proportion of CD20 positive cells in the parental P12 biopsy, virtually all PDX cells of VFN-T3 (derived from P11) stained CD20-positive. Despite expressing CD20, the genetic profile of VFN-T3 cells was similar to that of primary lymphoma cells (Fig. 2, Supplementary Fig. 13).

## Discussion

We implemented a complex genetic and histopathological screening approach to compare 15 newly derived PDX models of aggressive lymphomas with their respective primary lymphoma biopsies. The established PDX models comprised all major aggressive NHL subtypes, including DLBCL of the germinal center (GC) and non-GC immunophenotypes, double-hit DLBCL, transformed DLBCL, MCL, Burkitt lymphoma, PTCL, AITL, ALCL, ALK-positive, and ALCL, ALK-negative. The genetic comparison by WES confirmed that the PDXs retained most somatic mutations and copy number variants present in the primary lymphoma cells. This finding is in agreement with previously published data^6,8,52,53^. Moreover, the PDXs shared not only similar numbers of somatic mutations, but also similar allele frequencies of these mutations. Besides comparable mutational landscapes, similar profiles of predicted CNVs were found in PDXs compared to the respective primary lymphoma samples. The WES data thus confirmed that, from the genetic perspective, the majority of the established PDXs did not represent mere subclones of the original, more heterogeneous tumors, but maintained their genetic diversity. In most of the analyzed PDXs, the numbers of shared mutations were higher than the sums of N/D and N/U mutations. Higher proportions of N/D and N/U mutations were observed only in 3 (out of 13) analyzed PDXs, namely in the PDX tumors VFN-D6 (P2), VFN-M1 (P10), and VFN-T6 (P13). Across all analyzed PDX models (except for VFN-T6), the numbers of N/D mutations were markedly higher than the numbers of N/U mutations. Notably, most N/D mutations were not found in the primary lymphoma samples (i.e., allele frequency 0) and comprised nucleotide transitions. These observations suggest that the vast majority of N/D mutations were de novo acquired during sustained cell divisions of PDX cells in the murine organism, e.g., by the operating somatic hypermutation process, which is also consistent with previously published data^54,55^. On the other hand, most N/U mutations were plausibly “lost” because of relative underrepresentation of disease subclones with low-frequency bystander mutations during the propagation of the PDX tumors in murine organism^56^.

In contrast to the generally shared genetic profiles, the histopathologic analysis revealed several consistently observed phenotypic alterations of the PDX cells and PDX TME compared to the original lymphoma cells and original lymphoma TME, respectively. The morphology of most PDX models displayed more aggressive features than the original lymphoma cells. Higher proliferation rates by Ki-67 were observed in several PDX models compared to the corresponding primary lymphoma biopsies. These observations suggest more aggressive phenotypes of the engrafted PDX tumors compared to the respective primary lymphoma samples. Considering the overall genetic similarity between the PDXs and the original lymphoma cells, these differences might be explained by differences in the composition of the PDX TME.

Indeed, we confirmed absence of any human non-malignant cells (lymphocytes, macrophages) within the PDX tumors, even though unsorted primary samples (containing both lymphoma cells and non-malignant cells of the TME) were injected into NSG mice. Curiously, murine macrophages were not detected in the PDX TME either, even though they could be found in the murine spleen, liver, and bone marrow. Moreover, the PDX tumors had significantly lower MVD and MVA compared to the original lymph node biopsies. Importantly, only murine, not human vessels were detected in the PDX tumors. The lower MVD/MVA profiles might be a consequence of suboptimal stimulation of the murine sprouting angiogenesis with human vascular endothelial growth factor (VEGF) produced by lymphoma cells under hypoxia. The significantly lower MVD/MVA of murine origin may have large-scale implications for the results of preclinical studies with anti-angiogenic agents, or drugs based on passive accumulation in the tumor tissue, e.g., liposome-encapsulated formulations of cytostatics.

In addition to changes in the composition of the TME, immunophenotypic alterations of PDX cells were observed as well, including low expression of SOX11 (and to a lesser extent of cyclin D1) in both PDX models of MCL; a lack of expression of cytotoxic granules and changed expression of lymphoma-associated markers (CD20, CD3) in several T-NHL PDX models. Curiously, the expression of cyclin D1 appeared weaker (compared to the original lymphoma samples) in both PDX tumors of MCL in the manuscript by Zhang et al.^8^.

In general, PDX models are established upon engraftment of primary lymphoma samples in immunodeficient mice. Several techniques have been developed, including subrenal capsule implantation and intravenous, subcutaneous or intraperitoneal injections^5,6,8,57^. The omental tumor xenograft (OTM) model, developed by Burack et al., enabled xenotransplantation and study of some non-malignant cell populations including CD4 + T-cells^7^. It is, however, not clear, whether the OTM model enables effective serial retransplantations of the engrafted PDX cells.

The PDX models described in this study maintained the genetic profiles, but clearly lost the TME heterogeneity observed in the primary lymphoma samples from which they had been derived. It might be speculated that PDX models described in this study represent a selection of PDX models with the lowest dependence on human TME. Indeed, during the 10 years of our sustained effort to develop PDX models of various NHLs, the engraftment rates of primary lymphoma samples into NSG mice were ~25–30% (data not shown). It might be hypothesized that the primary lymphoma samples that depended on the non-malignant lymphoma TME components for their survival and proliferation did not engraft. On the other hand, even the established PDX cells could not be expanded ex vivo/in vitro (data not shown) thereby indirectly confirming their dependence on the in vivo type of growth. It can only be speculated, which of the in vivo factors contributed to the survival, engraftment, and growth of the successfully established PDX tumors. These might include cell-to-cell contact, hypoxia, metabolic requirements, or other factors.

In conclusion, PDX models do represent the most relevant tools currently available for various aspects of translational research including preclinical assessment of experimental treatment approaches. However, to avoid unwanted biases, the experiments conducted on murine PDX models of aggressive lymphomas should always take into consideration potential histopathological discrepancies between PDX models and primary lymphoma samples, as described in detail in this study.

## Supplementary information

Supplemental Data File

## Data Availability

All data generated or analyzed during this study are included in this published article [and its supplementary information files].
