# Supplementary material for: Complex genetic and histopathological study of 15 patient-derived xenografts of aggressive lymphomas
Source: Lab Invest. 2022 Apr 29;102(9):957–65. doi: 10.1038/s41374-022-00784-w (PMC9420679; doi:10.1038/s41374-022-00784-w)
Supplement: Supplementary file 1 — Supplemental Data File [file 41374_2022_784_MOESM1_ESM.docx]

**SUPPLEMENTAL DATA FILE**

**Complex genetic and histopathological study of 15 patient-derived xenografts of aggressive lymphomas**

**Running Title:** Patient-derived xenografts of aggressive lymphomas

**Authors:** Radek Jakša^1^, Jana Karolová^2,3^, Michael Svatoň^4^, Dmitry Kazantsev^2^, Martina Grajciarová^5^, Eva Pokorná^2^, Zbyněk Tonar^5^, Magdalena Klánová^2,3^, Lucie Winkowska^4^, Diana Maláriková^2,3^, Petra Vočková^2,3^, Kristina Forsterová^3^, Nicol Renešová^2^, Alexandra Dolníková^2^, Kristýna Nožičková^2^, Pavel Dundr^1^, Eva Froňková^4^, Marek Trněný^3^, and Pavel Klener^2,3*^

**Corresponding author*

| **Supplemental Table 1.** List of antibodies | | | | | | | |
| --- | --- | --- | --- | --- | --- | --- | --- |
| **Antibody** | **Clone** | **Dilution** | **Producer** | **Platform** | **Detection** | **Detected target** | **Lymphoma subtype** |
| CD45 | 2B11 + PD7/26 | 1:200 | Dako, Glostrup, Denmark | PT-link (Agilent, Santa Clara, CA, USA) | EnVision FLEX (Agilent) | Human leukocytes | all |
| Ki-67 | MIB-1 | 1:100 | Dako, Glostrup, Denmark | Ventana BenchMark ULTRA (Roche, Basel, Switzerland) | OptiView | Proliferation rate | all |
| MYC | Y69 | 1:20 | Abcam, Cambridge, United Kingdom | Ventana BenchMark ULTRA (Roche, Basel, Switzerland) | OptiView | transcription factor MYC | all |
| human CD31 | JC70A | 1:50 | Dako, Glostrup, Denmark | PT-link (Agilent, Santa Clara, CA, USA) | EnVision FLEX (Agilent) | Human vessels | all |
| mouse CD31 | SP38 | 1:50 | Termofisher Scientific, MA, USA | PT-link (Agilent, Santa Clara, CA, USA) | EnVision FLEX (Agilent) | Murine vessels | all |
| human CD68 | PG-M1 | 1:25 | Dako, Glostrup, Denmark | PT-link (Agilent, Santa Clara, CA, USA) | EnVision FLEX (Agilent) | Human macrophages | all |
| mouse CD68 | SP38 | 1:50 | Termofisher Scientific, Waltham, MA, USA | PT-link (Agilent, Santa Clara, CA, USA) | EnVision FLEX (Agilent) | Murine macrophages | all |
| CD3 | LN10 | 1:500 | Novocastra, Leica Biosystems, Wetzlar, Germany | PT-link (Agilent, Santa Clara, CA, USA) | EnVision FLEX (Agilent) | Human lymphocytes | all |
| CD4 | SP35 | 1:100 | DCS, Innovative Diagnostik-Systeme, Germany | PT-link (Agilent, Santa Clara, CA, USA) | EnVision FLEX (Agilent) | Helper T-cells | all |
| CD8 | C8/144B | 1:100 | Dako, Glostrup, Denmark | PT-link (Agilent, Santa Clara, CA, USA) | EnVision FLEX (Agilent) | Cytotoxic T-cells | all |
| CD56 | 123C3.D5 | 1:25 | Zeta Corporation, Sierra Madre, CA, USA | PT-link (Agilent, Santa Clara, CA, USA) | EnVision FLEX (Agilent) | Human natural killer cells | all |
| CD20 | L26 | 1:400 | Dako, Glostrup, Denmark | PT-link (Agilent, Santa Clara, CA, USA) | EnVision FLEX (Agilent) | Human B lymphocytes | DLBCL, MCL, FL, BL |
| BCL2 | 124 | 1:100 | Dako, Glostrup, Denmark | PT-link (Agilent, Santa Clara, CA, USA) | EnVision FLEX (Agilent) | Anti-apoptotic BCL2 protein | DLBCL, MCL, FL, BL |
| BCL6 | PG-B6p | 1:10 | Dako, Glostrup, Denmark | Ventana BenchMark ULTRA (Roche, Basel, Switzerland) | OptiView | Transcription factor BCL6 | DLBCL, AITL |
| CD10 | 56C6 | 1:50 | Novocastra, Leica Biosystems, Wetzlar, Germany | Ventana BenchMark ULTRA (Roche, Basel, Switzerland) | OptiView | Germinal center cells | DLBCL, AITL |
| IRF4 / MUM1 | MUM1p | 1:100 | Dako, Glostrup, Denmark | PT-link (Agilent, Santa Clara, CA, USA) | EnVision FLEX (Agilent) | Transcription factor IRF4 | DLBCL |
| Cyclin D1 | SP4 | 1:200 | Dako, Glostrup, Denmark | Ventana BenchMark ULTRA (Roche, Basel, Switzerland) | OptiView | Cyclin D1 | MCL |
| SOX11 | MRQ-58 | 1:200 | Cell Marque, Rocklin, CA, USA | PT-link (Agilent, Santa Clara, CA, USA) | EnVision FLEX (Agilent) | Transcription factor SOX11 | MCL |
| CD5 | 4C7 | 1:400 | Novocastra, Leica Biosystems, Wetzlar, Germany | Ventana BenchMark ULTRA (Roche, Basel, Switzerland) | OptiView | T cells | MCL, ALCL |
| ALK | 5A4 | 1:100 | Zytomed systems, Berlin, Germany | PT-link (Agilent, Santa Clara, CA, USA) | EnVision FLEX (Agilent) | Anaplastic lymphoma kinase ALK | ALCL |
| CD23 | 1B12 | 1:100 | Novocastra, Leica Biosystems, Wetzlar, Germany | PT-link (Agilent, Santa Clara, CA, USA) | EnVision FLEX (Agilent) | Follicular dendritic cells | ALCL |
| PD-1 | NAT-105 | 1:25 | Cell Marque, Rocklin, CA, USA | PT-link (Agilent, Santa Clara, CA, USA) | EnVision FLEX (Agilent) | Programmed death 1 receptor | AITL |
| CD7 | CBC.37 | 1:200 | Dako, Glostrup, Denmark | Ventana BenchMark ULTRA (Roche, Basel, Switzerland) | OptiView | T cells | PTCL |
| Granzyme B | 11F1 | 1:40 | Novocastra, Leica Biosystems, Wetzlar, Germany | PT-link (Agilent, Santa Clara, CA, USA) | EnVision FLEX (Agilent) | Cytotoxic granules of T cells | PTCL |
| Perforin | 5B10 | 1:40 | Leica microsystems | Ventana BenchMark ULTRA (Roche, Basel, Switzerland) | OptiView | Cytotoxic granules of T cells | PTCL |
| TIA-1 | 2G9A10F5 | 1:20 | BioGenex, Fremont, CA, USA | Ventana BenchMark ULTRA (Roche, Basel, Switzerland) | OptiView | Cytotoxic granules of T cells | PTCL |
| **Abbreviations:** AITL= angioimmunoblastic T-cell lymphoma; ALCL= anaplastic large cell lymphoma; BL= Burkitt lymphoma; DLBCL= diffuse large B-cell lymphoma; FL= follicular lymphoma; MCL= mantle cell lymphoma; PTCL= peripheral T-cell lymphoma | | | | | | | |

| **Supplemental Table 2.** Quantitative histological parameters | | | |
| --- | --- | --- | --- |
| **Abbreviation and unit** | **Quantitative parameter** | **Measurement procedure** | **Biological significance** |
| MVD, microvessel density (vascular profiles/mm^2^) | The quantity of the CD31-positive microvessel profiles per area of the section | Unbiased counting frame for counting vessel profiles | A two-dimensional quantitative estimate of the intratumoral microvascular bed. In randomly oriented sections, this parameter highly correlates with the three-dimensional length density of microvessels |
| MVA, microvascular area (per mill) | Area fraction of CD31-positive microvessel profiles within the tumor | Stereological point grid for estimating area of vessel profiles | A two-dimensional quantitative estimate of the degree of anomalous intratumoral vessels and pathophysiological alterations |

| **Supplemental Table 3.** Quantification of intratumoral microvessel density and area | | | | | | | | |
| --- | --- | --- | --- | --- | --- | --- | --- | --- |
| **Patient** | **Slide No.** | **MVD (vascular profiles / mm^2^)** | **MVA (‰)** |  | **Patient** | **Slide No.** | **MVD (vascular profiles / mm^2^)** | **MVA (‰)** |
| **P3** | 1 | 161.3 | 11.5 |  | **P5** | 1 | 95.4 | 19.4 |
|  | 2 | 140.3 | 31.9 |  |  | 2 | 89.8 | 23.3 |
|  | 3 | 158.5 | 24.7 |  |  | 3 | 102.4 | 20.8 |
|  | 4 | 109.4 | 13.5 |  |  | 4 | 89.8 | 23.3 |
|  | 5 | 143.1 | 21.2 |  |  | 5 | 136.0 | 31.3 |
| **Average** | | **142.5** | **20.6** |  | **Average** | | **102.7** | **23.6** |
| **SD** | | **20.7** | **8.3** |  | **SD** | | **19.3** | **4.6** |
| **VFN-D1** | 1 | 78.5 | 2.4 |  | **VFN-D5** | 1 | 64.5 | 5.2 |
|  | 2 | 106.6 | 2.8 |  |  | 2 | 63.1 | 6.6 |
|  | 3 | 85.6 | 2.4 |  |  | 3 | 56.1 | 9.4 |
|  | 4 | 60.3 | 2.8 |  |  | 4 | 68.7 | 3.5 |
|  | 5 | 58.9 | 2.1 |  |  | 5 | 85.6 | 10.1 |
| **Average** | | **78.0** | **2.5** |  | **Average** | | **67.6** | 6.9 |
| **SD** | | **19.7** | **0.3** |  | **SD** | | **11.0** | **2.8** |
| **Ratio patient / PDX** | | 2.1 x | 8.6 x |  | **Ratio patient / PDX** | | 1.5 x | 3.4 x |
|  |  |  |  |  |  |  |  |  |
| **P9** | 1 | 244.0 | 18.1 |  | **P10** | 1 | 171.1 | 37.2 |
|  | 2 | 274.9 | 23.3 |  |  | 2 | 151.5 | 51 |
|  | 3 | 280.5 | 27.1 |  |  | 3 | 120.6 | 38.2 |
|  | 4 | 304.3 | 23.6 |  |  | 4 | 165.5 | 63.9 |
|  | 5 | 280.5 | 21.5 |  |  | 5 | 145.9 | 46.2 |
| **Average** | | **276.8** | **22.7** |  | **Average** | | **150.9** | **47.3** |
| **SD** | | **21.6** | **3.3** |  | **SD** | | **19.8** | **10.9** |
| **VFN-M5R1** | 1 | 91.2 | 5.9 |  | **VFN-M10** | 1 | 102.4 | 4.5 |
|  | 2 | 120.6 | 7.3 |  |  | 2 | 71.5 | 9.2 |
|  | 3 | 102.4 | 6.6 |  |  | 3 | 74.3 | 6.9 |
|  | 4 | 109.4 | 5.6 |  |  | 4 | 108 | 11.6 |
|  | 5 | 99.6 | 4.2 |  |  | 5 | 131.8 | 10.4 |
| **Average** | | **103.8** | **5.9** |  | **Average** | | **97.6** | **8.5** |
| **SD** | | **11.1** | **1.2** |  | **SD** | | **25.1** | **2.8** |
| **Ratio patient / PDX** | | 2.7 x | 3.8 x |  | **Ratio patient / PDX** | | 1.5 x | 5.6 x |

**Supplemental Table 4.** A complete list of filtered variants and gene lists of frequently and recurrently mutated genes in DLBCL, MCL, T-NHL, and BL, as well as pre-assembled CNV gene list can be downloaded from the following URL address: <https://doi.org/10.5281/zenodo.6035345>

**Supplemental Table 4 Legend:** A complete list of variants which passed filtering described in supplemental methods, that were found in both PDX model sample and patient’s sample from which it was derived (S1A) and variants which were newly detected (N/D) (S1B) or newly undetected (N/U) (S1C) during PDX model derivation. Gene lists for filtration of variants in genes of special interest are included in the table (S1D). Gene list for filtration of CNV changes in genes of special interest is included in the table (S1E). Chr- Chromosome, REF- Reference allele, ALT- Alternative allele, AA change- Amino acid change, Patient AF- Allele frequency in the patient’s sample, Patient Depth – Read depth in patient’s sample, PDX AF- Allele frequency in PDX model sample, PDX Depth - Read depth in PDX sample, DLBCL - Diffuse Large B-cell lymphoma, MCL - Mantle Cell Lymphoma, TCL - T-cell lymphoma, BL – Burkitt Lymphoma, and CNV - Copy Number Variation.

| **Supplemental Table 5.** Median allele frequencies of shared mutations in PDX models and primary lymphoma samples | | |
| --- | --- | --- |
|  | **Median AF of shared mutations in PDXs** | **Median AF of shared mutations in patient samples** |
| All | 0.39 | 0.27 |
| DLBCL | 0.44 | 0.36 |
| MCL | 0.45 | 0.44 |
| T-NHL | 0.41 | 0.27 |
| BL* | 0.42 | 0.44 |
| VFN-D3 vs P1 | 0.43 | 0.38 |
| VFN-D6 vs P2 | 0.44 | 0.26 |
| VFN-D1 vs P3 | 0.47 | 0.38 |
| VFN-D4 vs P4 | 0.44 | 0.3 |
| VFN-D5 vs P5 | 0.43 | 0.39 |
| VFN-D20 vs P7 | 0.47 | 0.48 |
| VFN-B3 vs P8 | 0.42 | 0.44 |
| VFN-M5R1 vs P9 | 0.45 | 0.43 |
| VFN-M1 vs P10 | 0.47 | 0.44 |
| VFN-T3 vs P11 | 0.2 | 0.1 |
| VFN-T6 vs P13 | 0.42 | 0.09 |
| VFN-T5 vs P14 | 0.45 | 0.42 |
| VFN-T4 vs P15 | 0.38 | 0.16 |
| **Legend:** A. Median allele frequencies (AF) of shared mutations in PDX models and primary lymphoma samples; BL= Burkitt lymphoma; DLBCL= diffuse large B-cell lymphoma; MCL= mantle cell lymphoma; P1-P15= patient primary lymphoma samples; T-NHL= T-cell non-Hodgkin lymphomas; VFN= PDX models derived from primary lymphoma samples P1-P15; * only 1 PDX model (VFN-B3) | | |

**
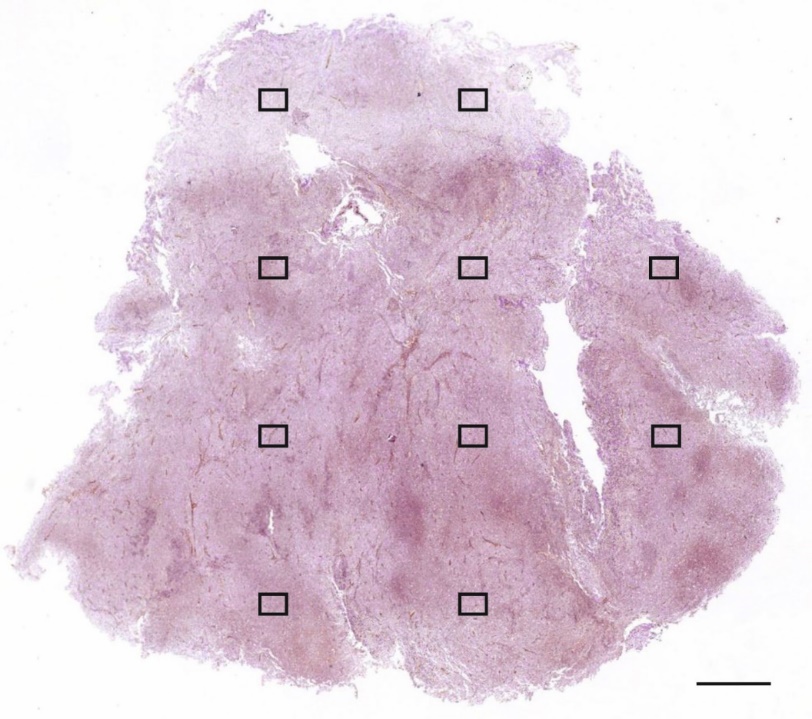
**

**Supplemental Figure 1.** Uniform sampling of microphotographs demonstrated by rectangles. Ten microphotographs sampled from the intratumoral reference space in each slide were used for quantification. Scale bar 1000 µm.

**
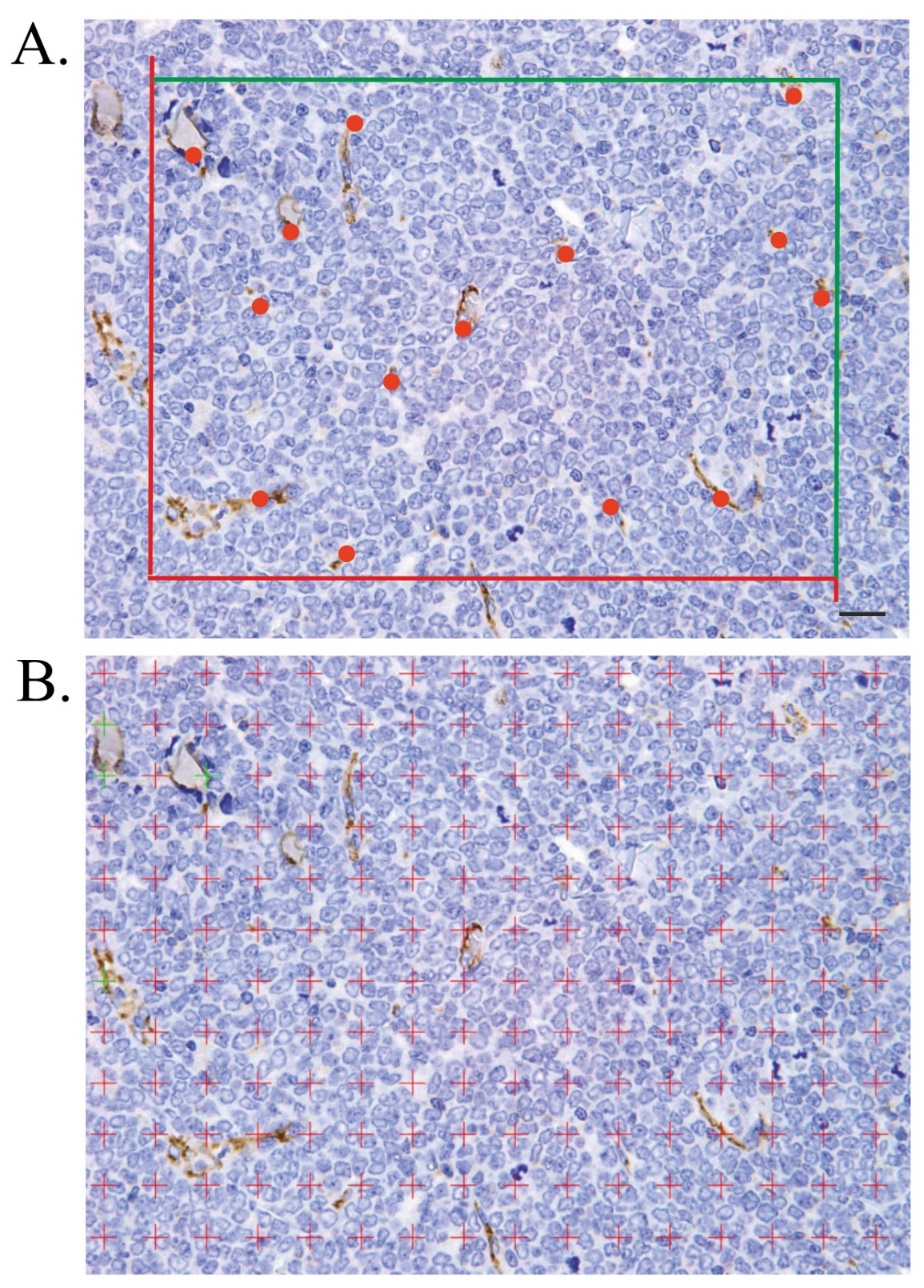
**

**Supplemental Figure 2.** Quantification of intratumoral microvessel density MVD (A) and microvessel area MVA (B) of the CD31-positive vascular profiles (stained brown) in human biopsies and corresponding mouse PDX models. Unbiased counting frames were used to quantify MVD (A). Stereological point grids and Cavalieri principle were used for MVA estimates (B). Scale bar 20 µm (A, B).


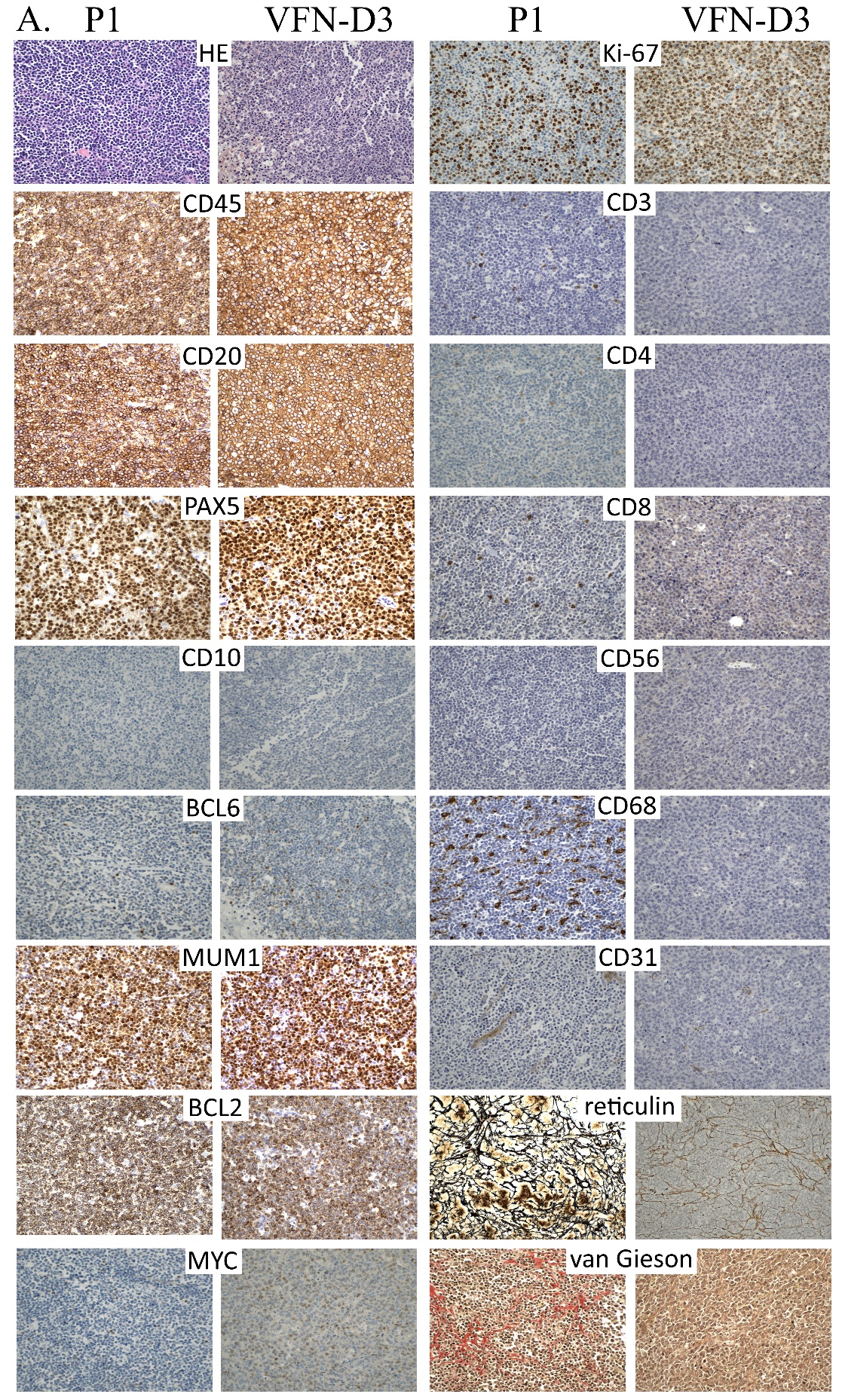

**Supplemental Figure 3.** Immunohistochemistry analysis of the PDX tumor VFN-D3 and the corresponding lymphoma biopsy (P1)

**Legend for Supplemental Figure 3:** A. Representative photos of immunohistochemistry analysis of primary lymphoma sample (P1) and the respective PDX model (VFN-D3); B. Evaluation of immunohistochemistry.

**
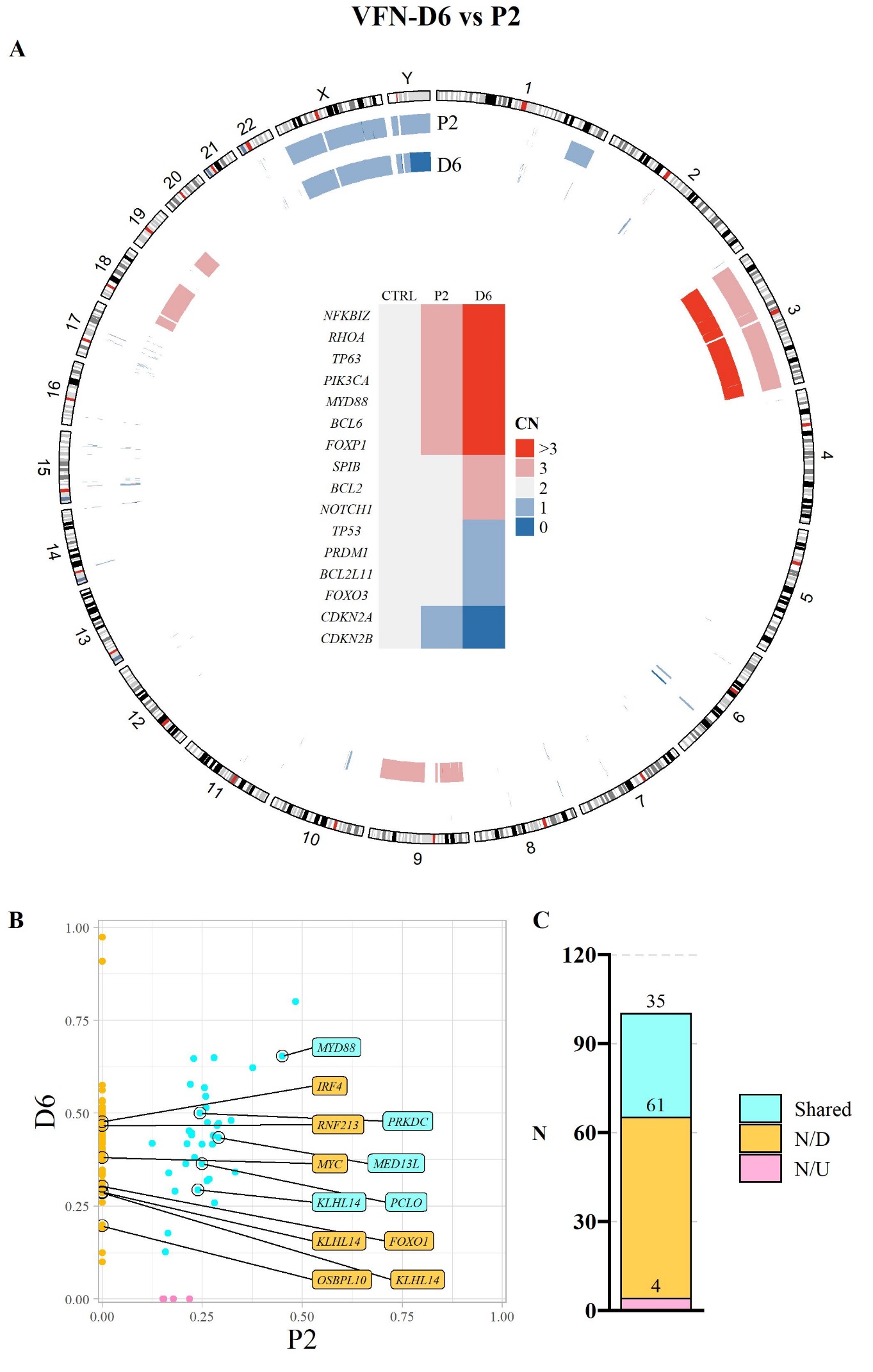
**

**
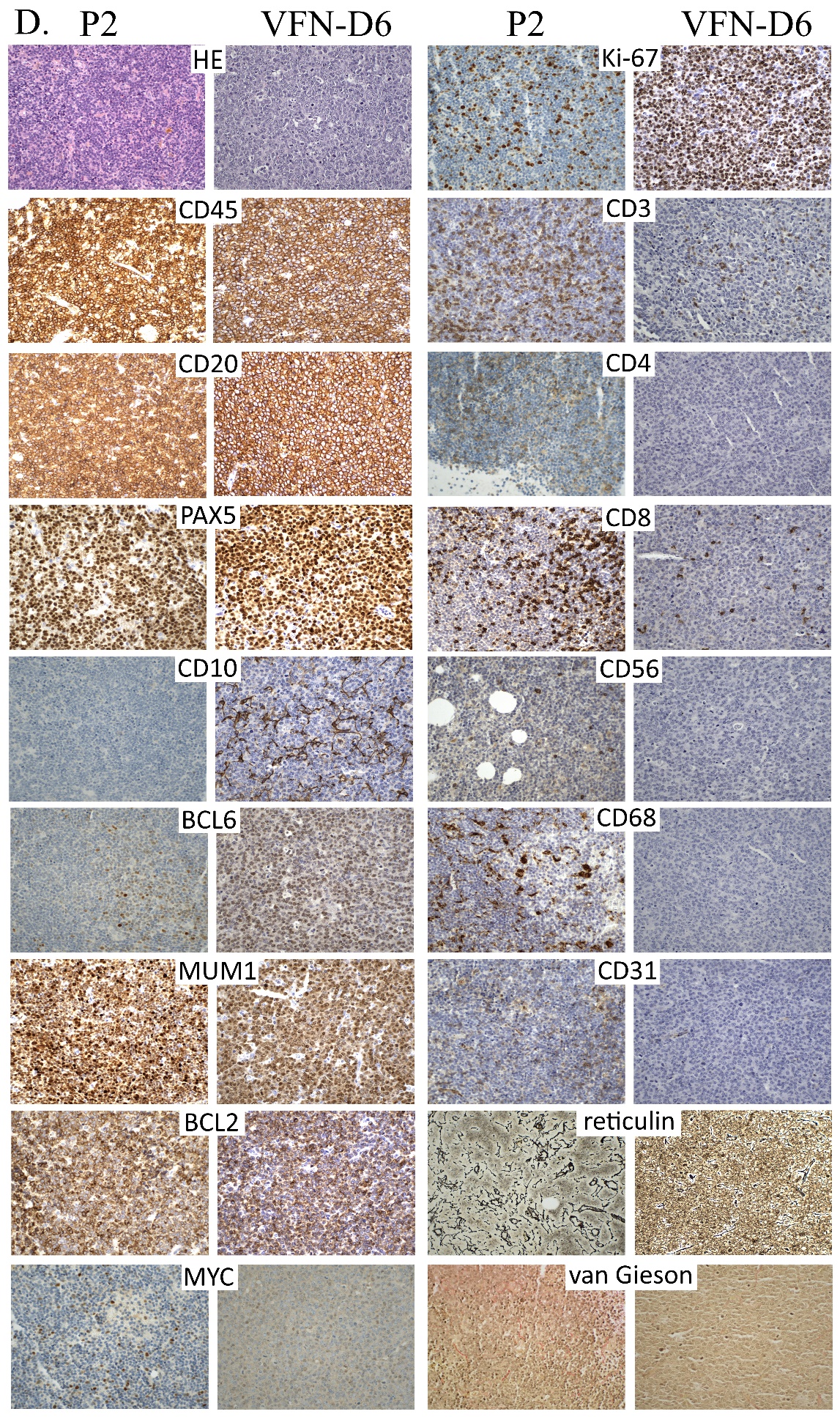
**

**Supplemental Figure 4.** Genetic and immunohistochemistry analysis of the PDX tumor VFN-D6 and the corresponding lymphoma biopsy (P2)

**
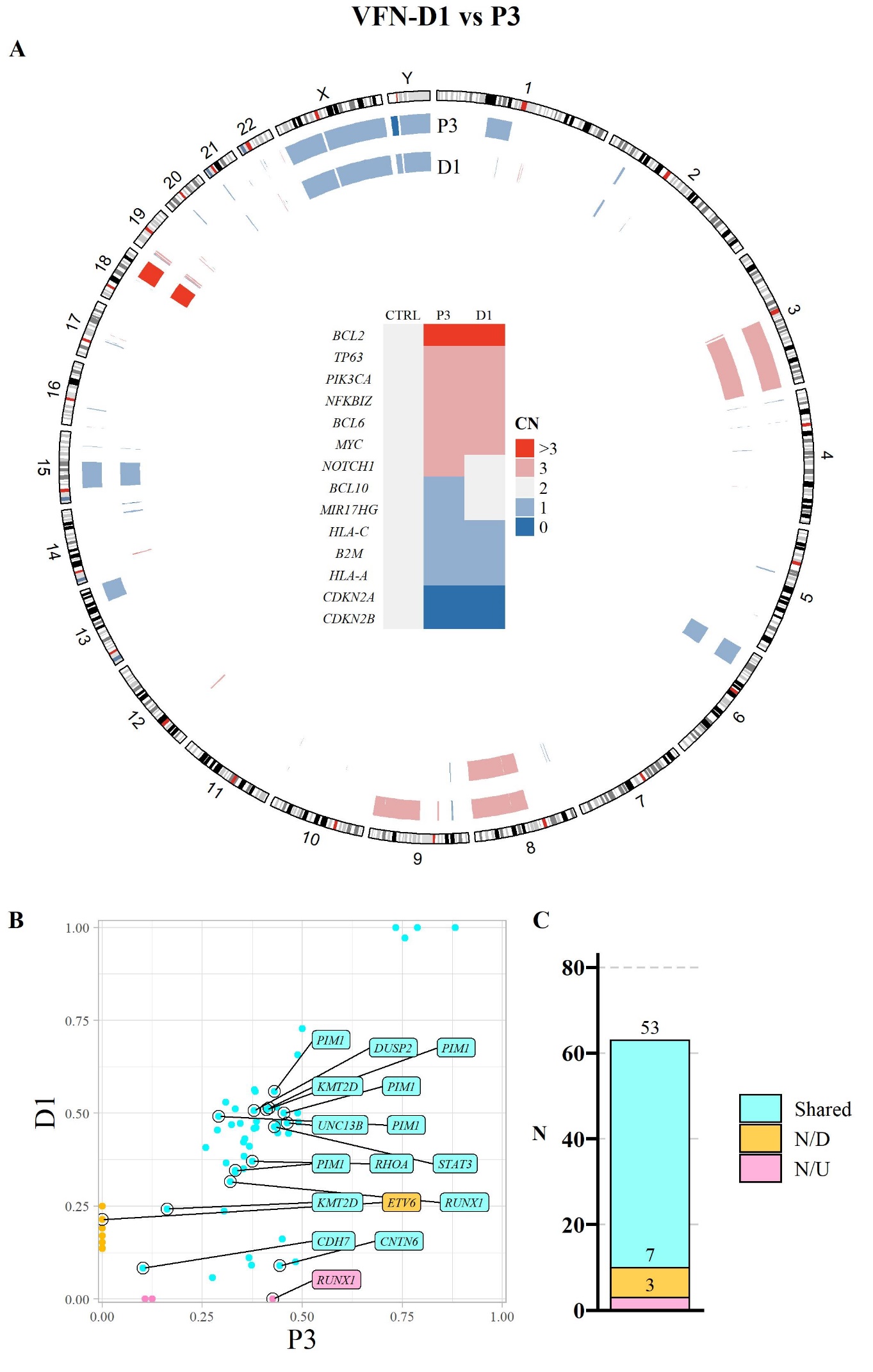
**

**
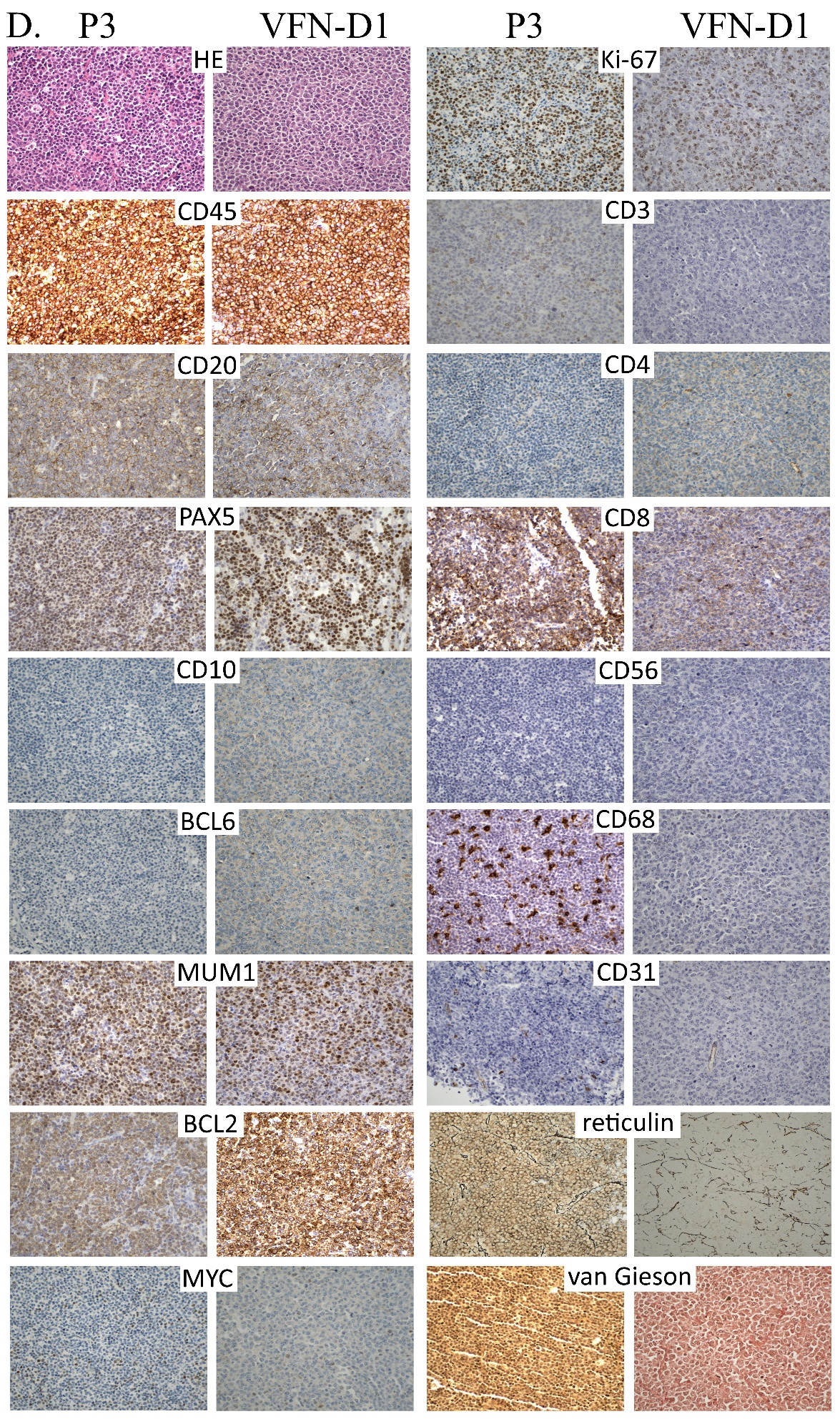
**

**Supplemental Figure 5.** Genetic and immunohistochemistry analysis of the PDX tumor VFN-D1 and the corresponding lymphoma biopsy (P3)

**
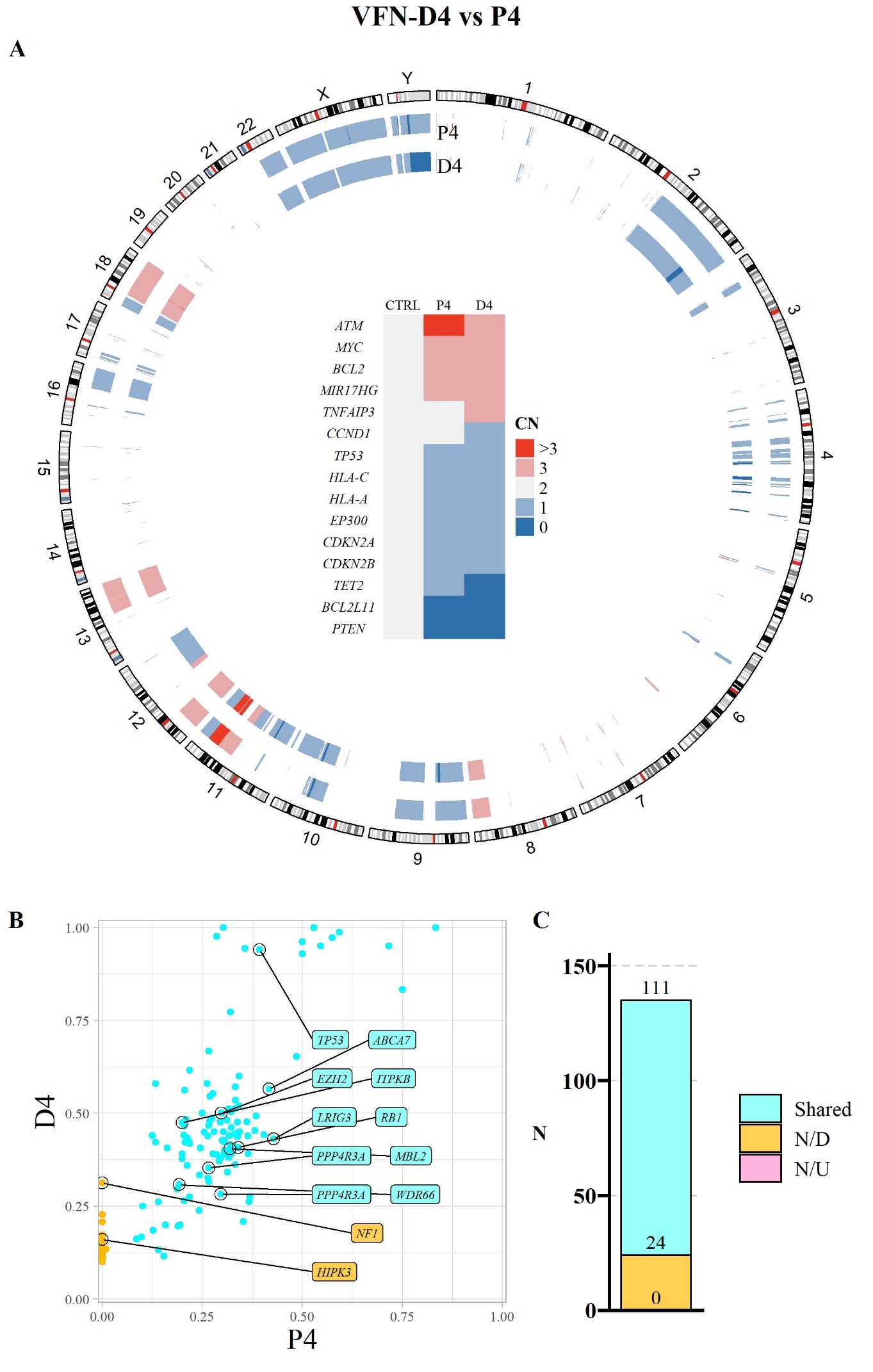
**

**
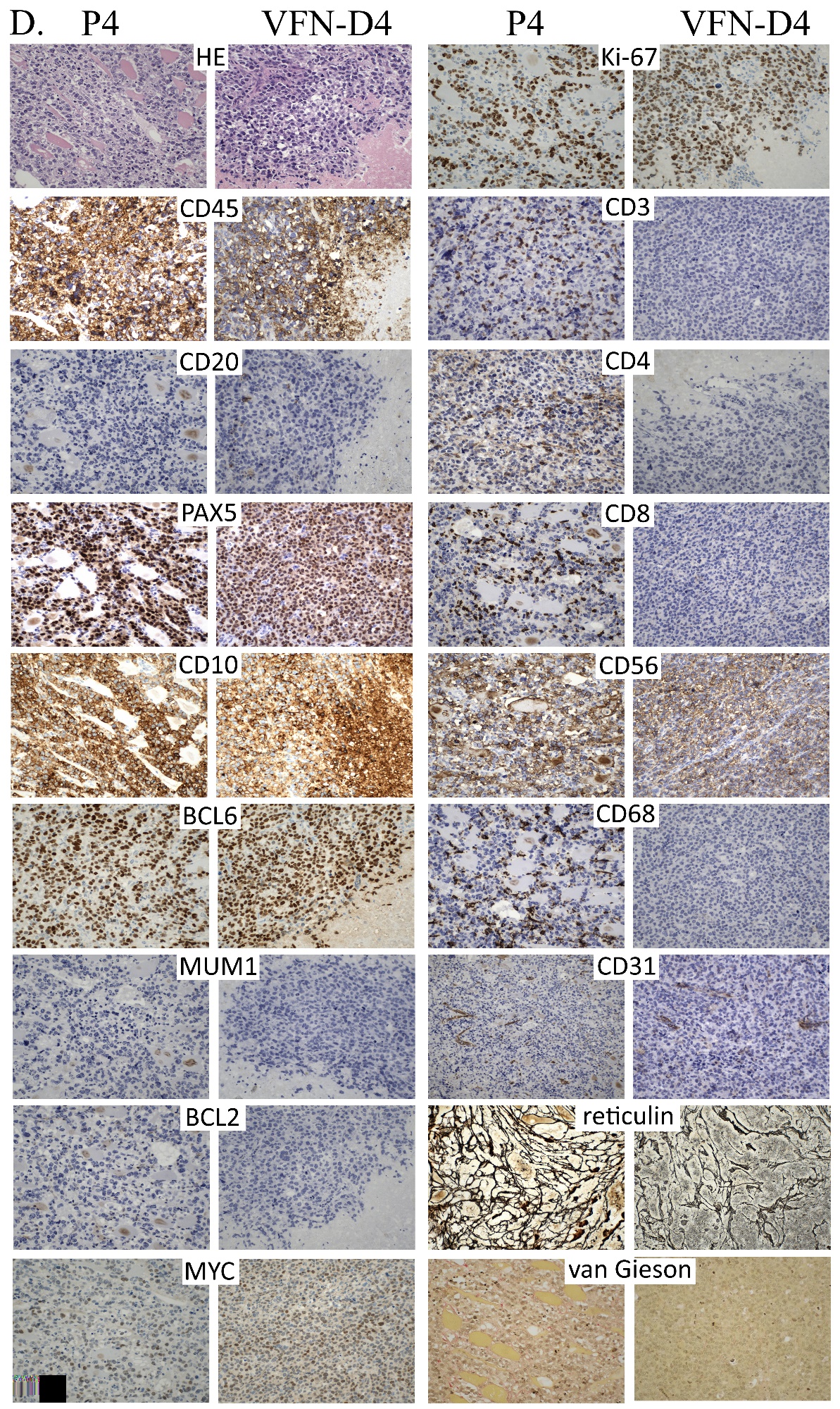
**

**Supplemental Figure 6.** Genetic and immunohistochemistry analysis of the PDX tumor VFN-D4 and the corresponding lymphoma biopsy (P4)

**
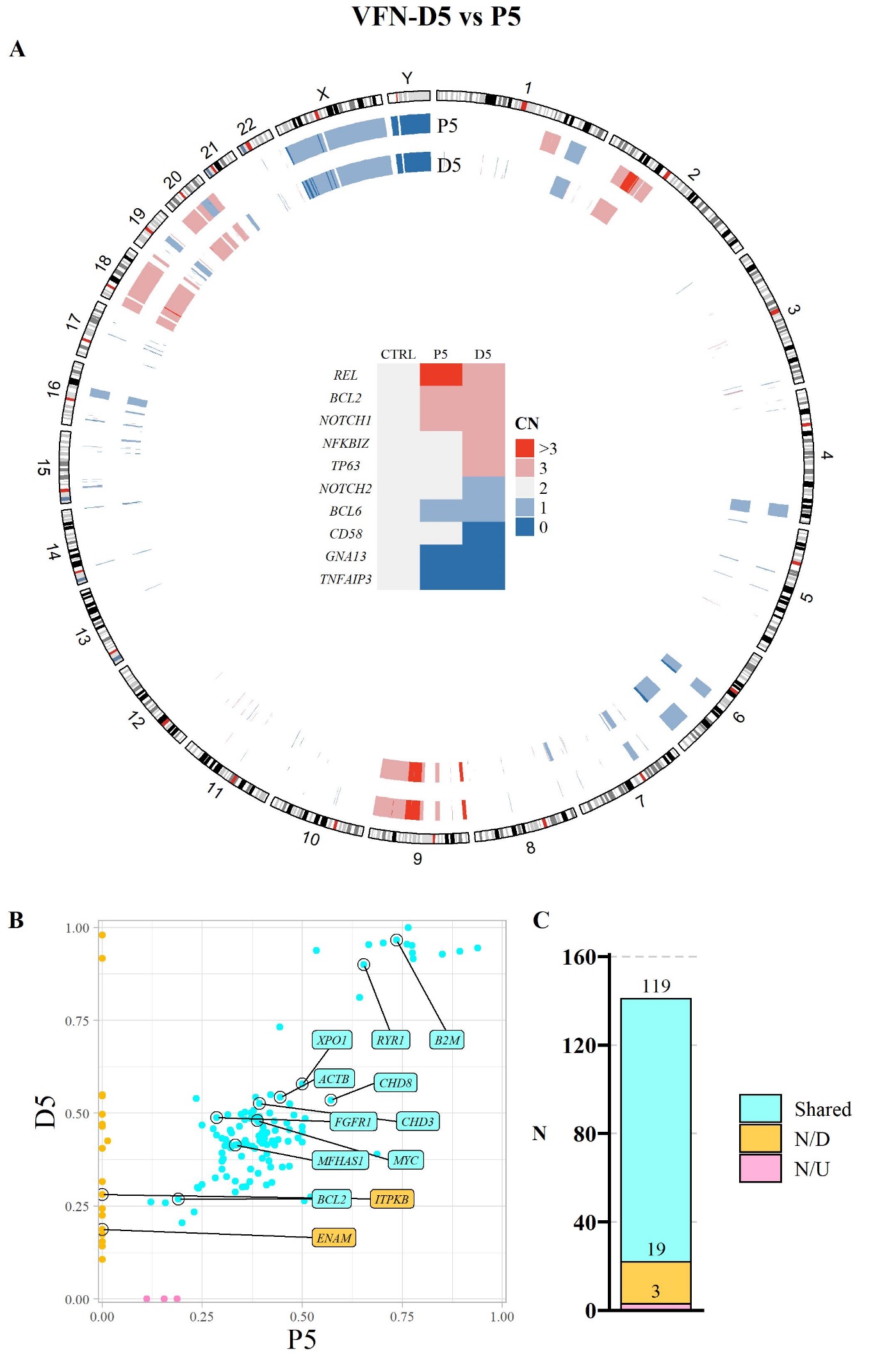
**

**
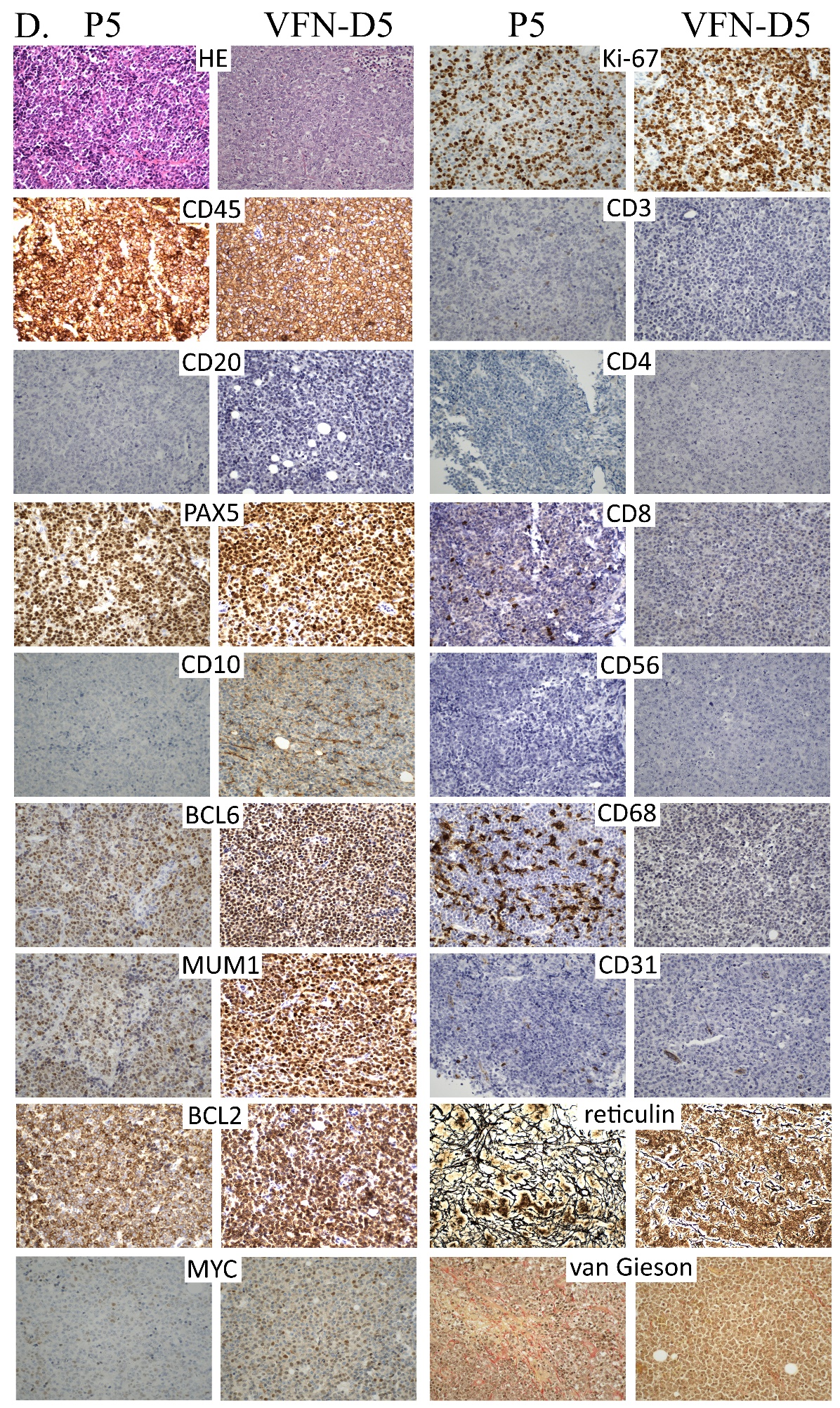
**

**Supplemental Figure 7.** Genetic and immunohistochemistry analysis of the PDX tumor VFN-D5 and the corresponding lymphoma biopsy (P5)

**
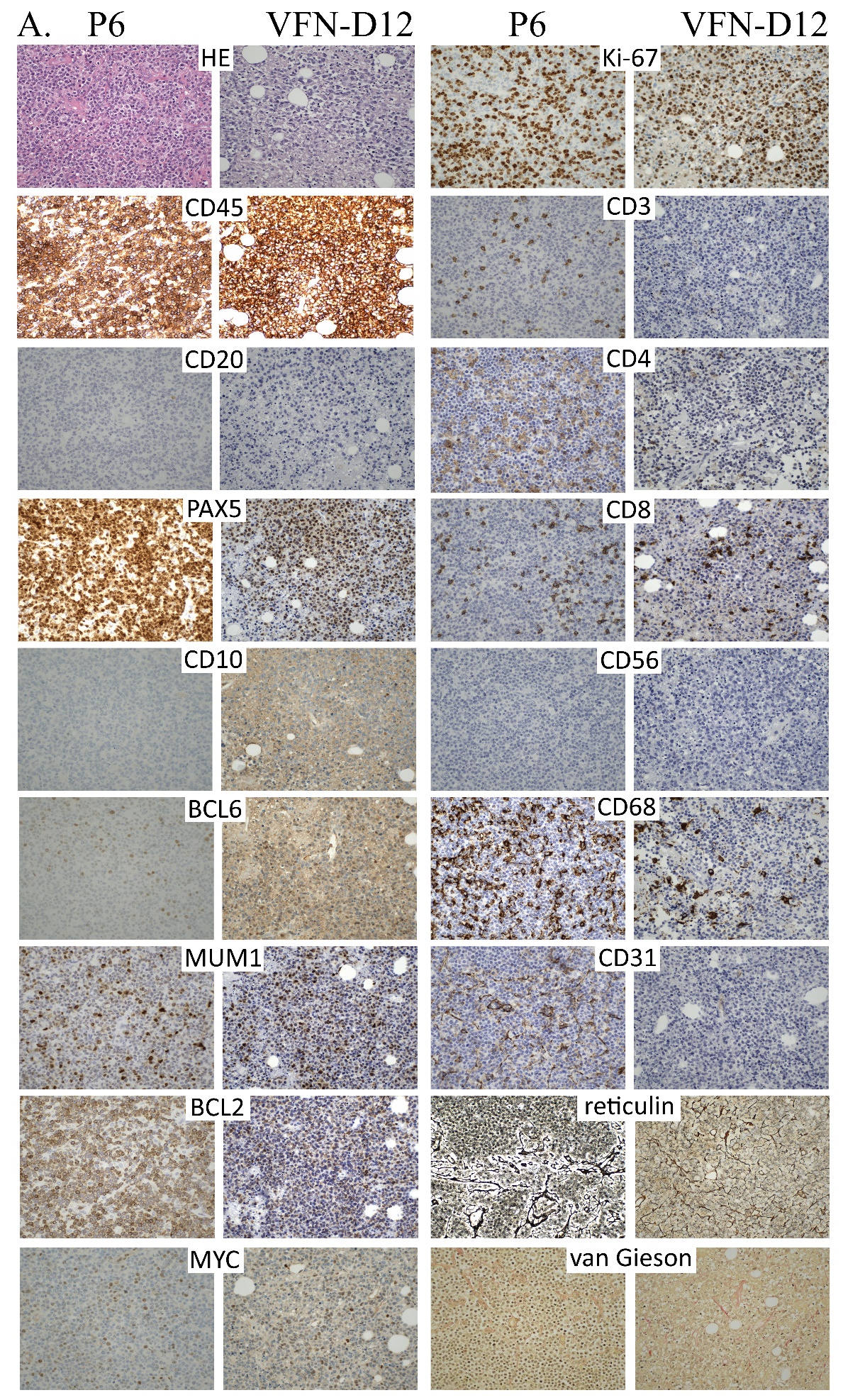
**

**Supplemental Figure 8.** Immunohistochemistry analysis of the PDX tumor VFN-D12 and the corresponding lymphoma biopsy (P6)


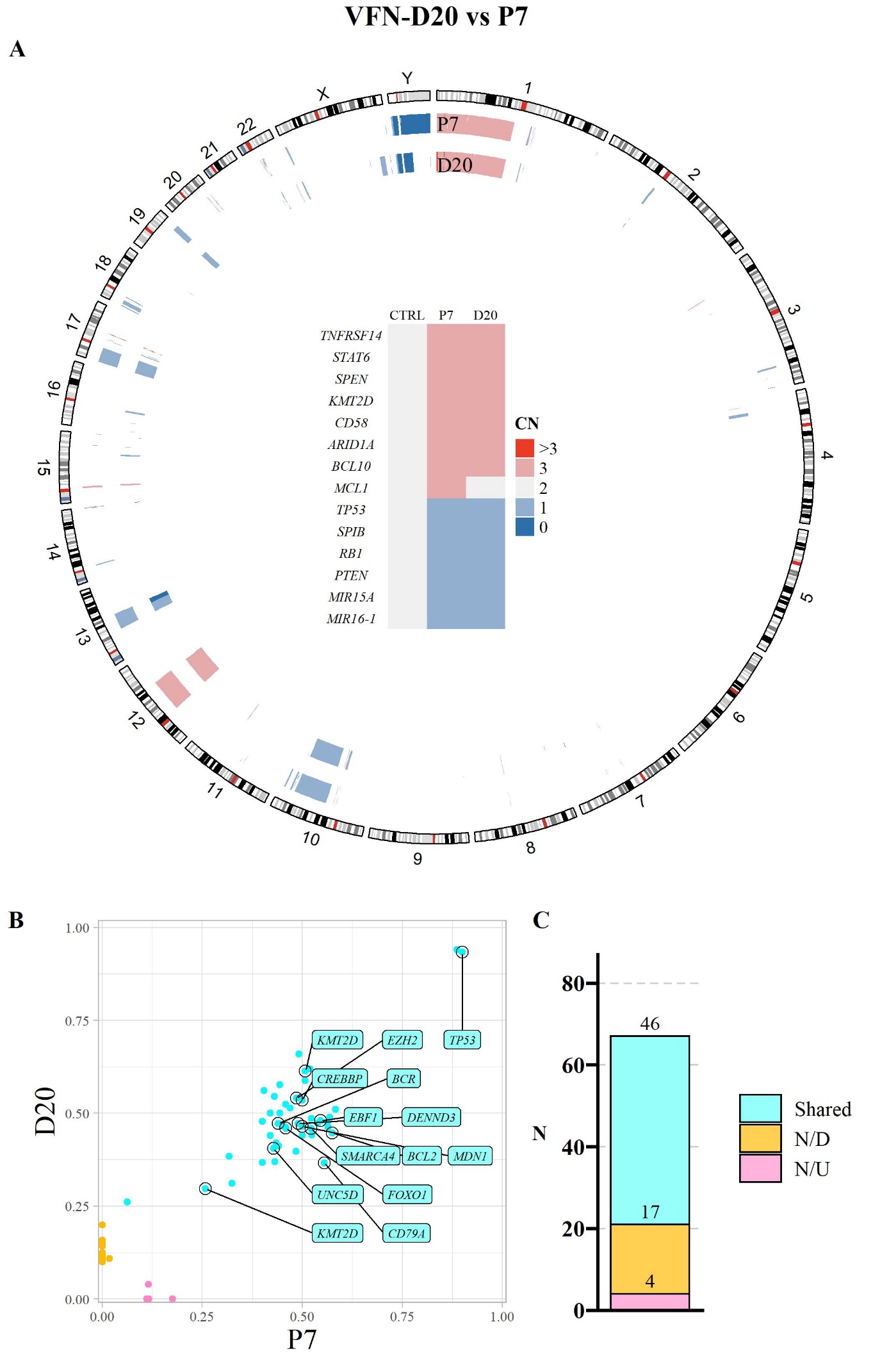


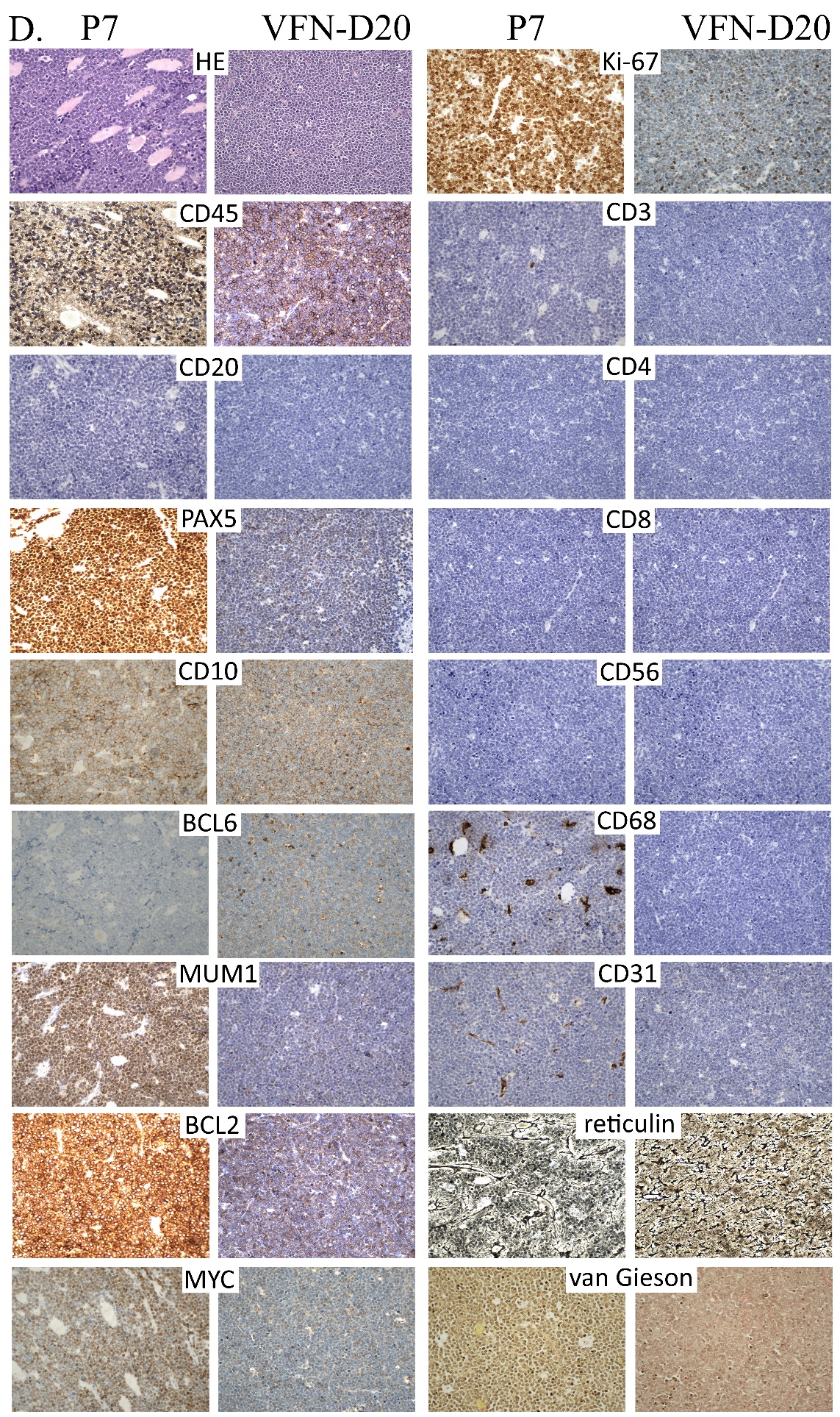

**Supplemental Figure 9.** Genetic and immunohistochemistry analysis of the PDX tumor VFN-D20 and the corresponding lymphoma biopsy (P7)


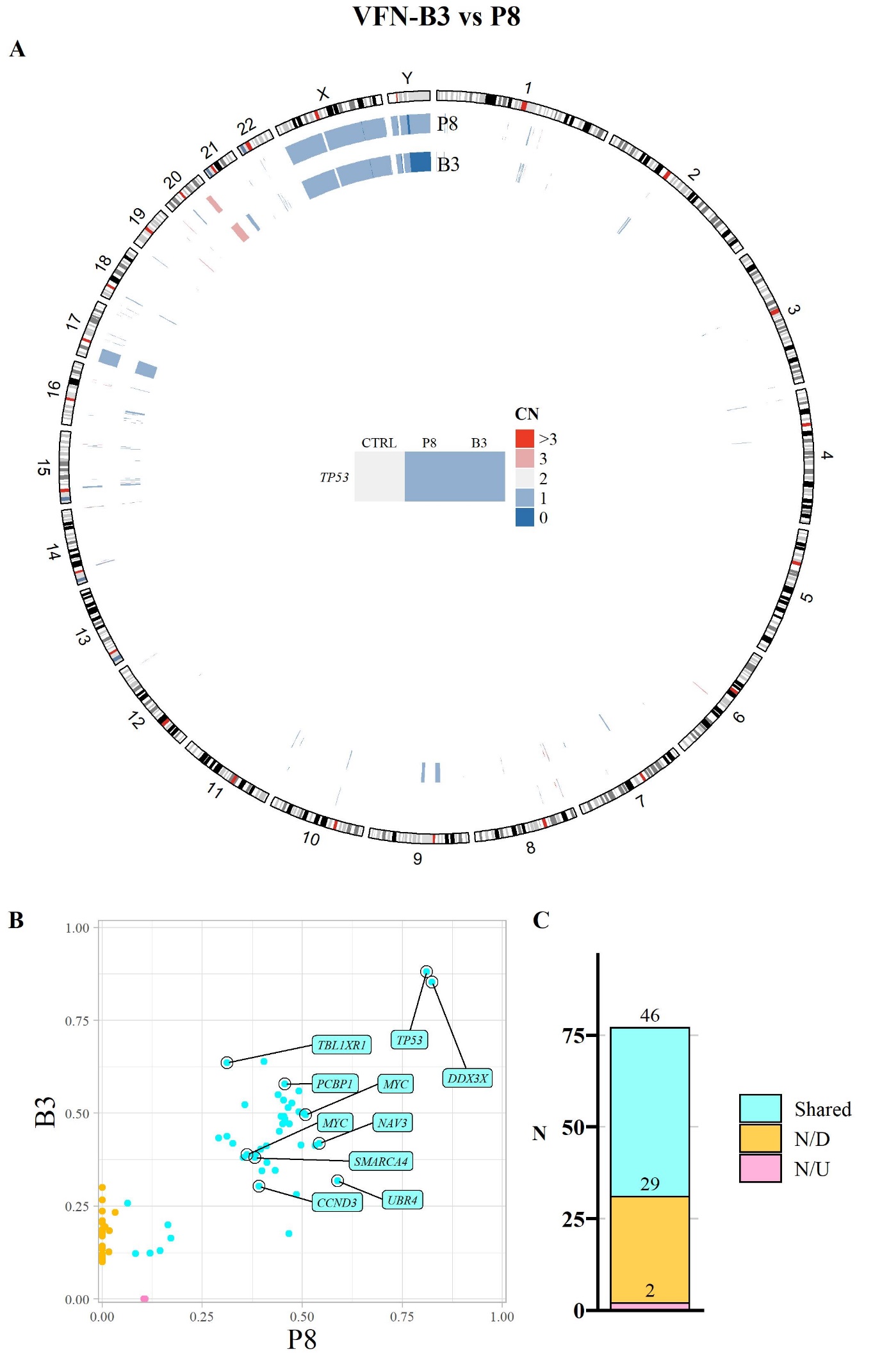


**
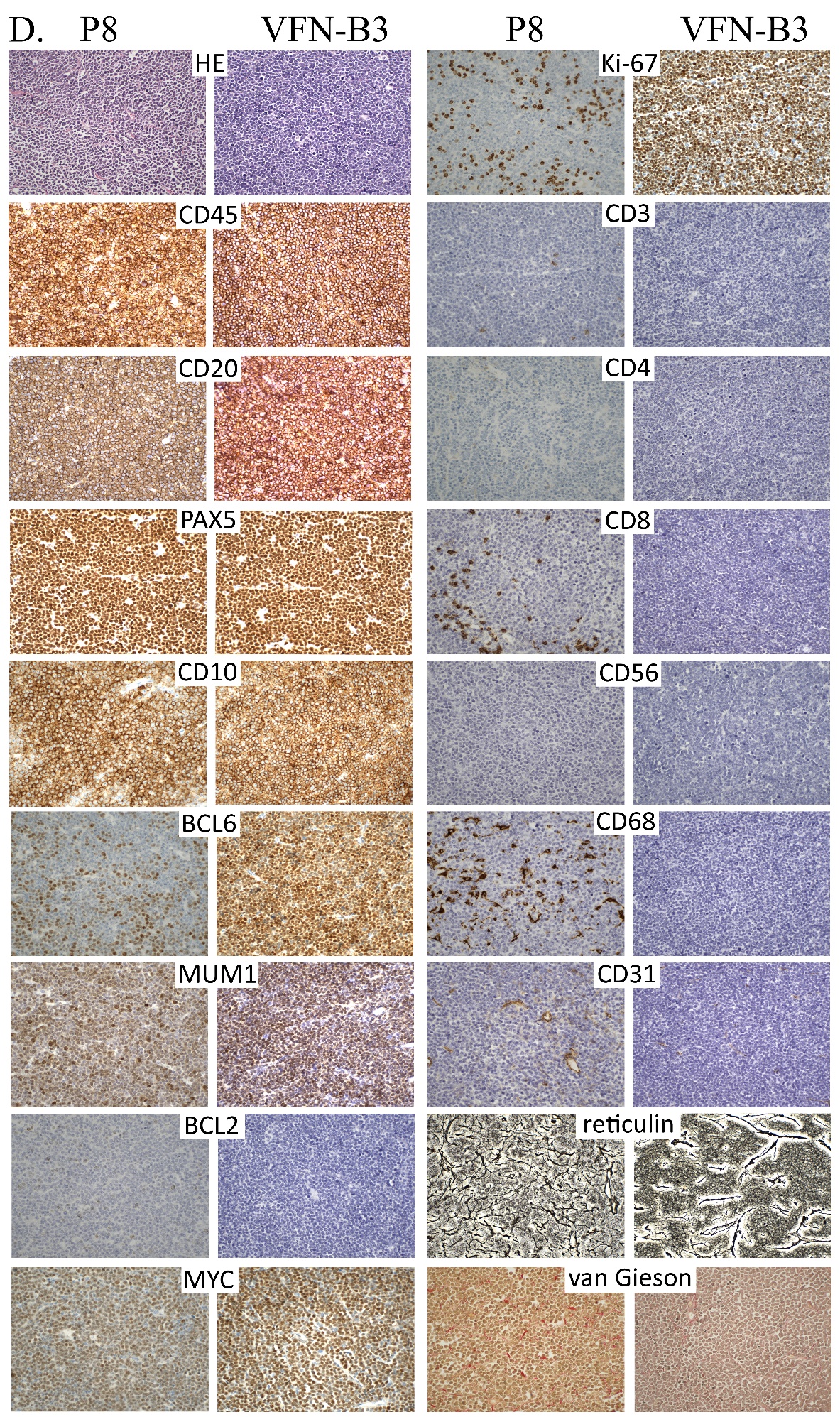
**

**Supplemental Figure 10.** Genetic and immunohistochemistry analysis of the PDX tumor VFN-B3 and the corresponding lymphoma biopsy (P8)

**
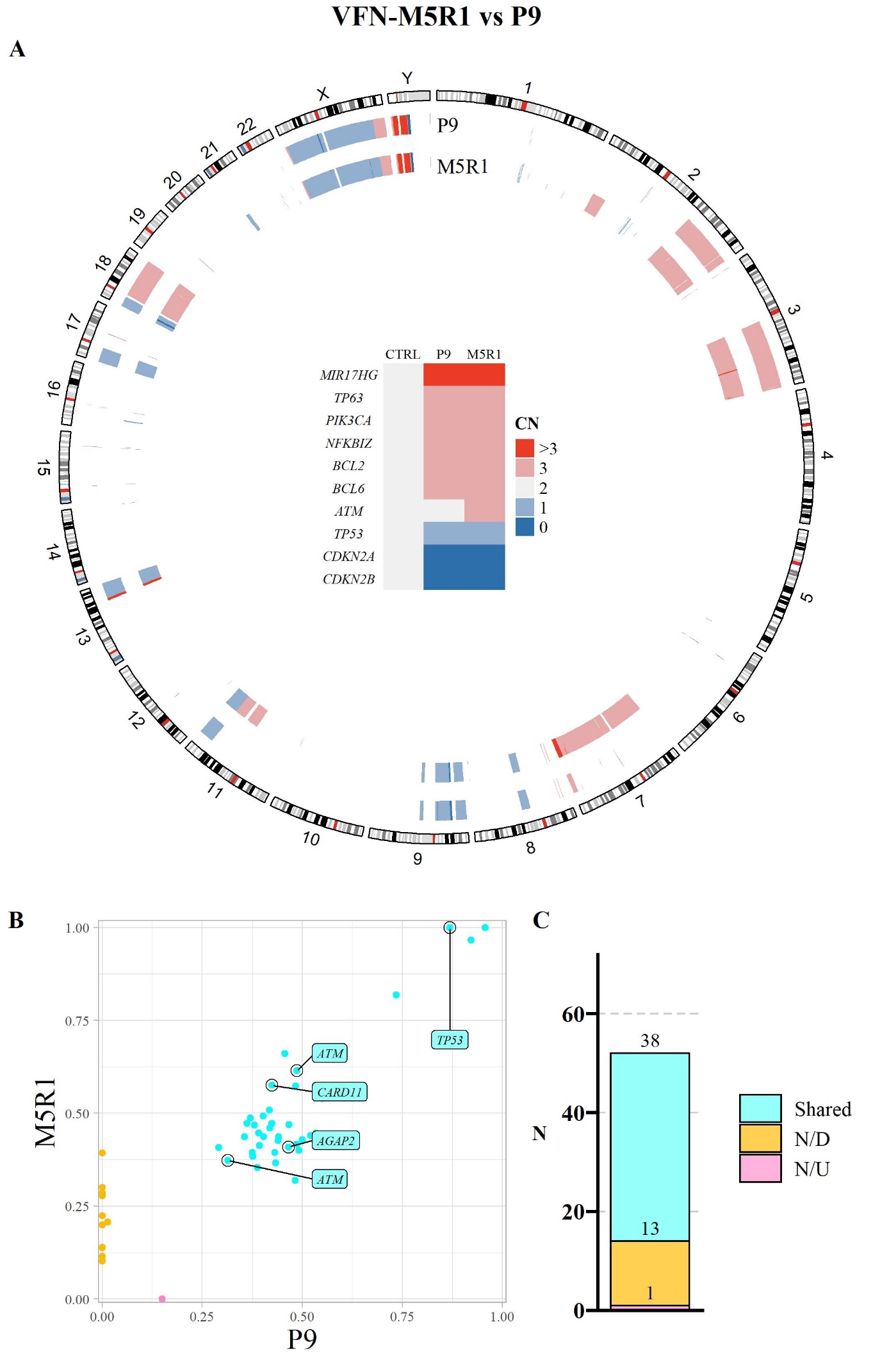
**

**
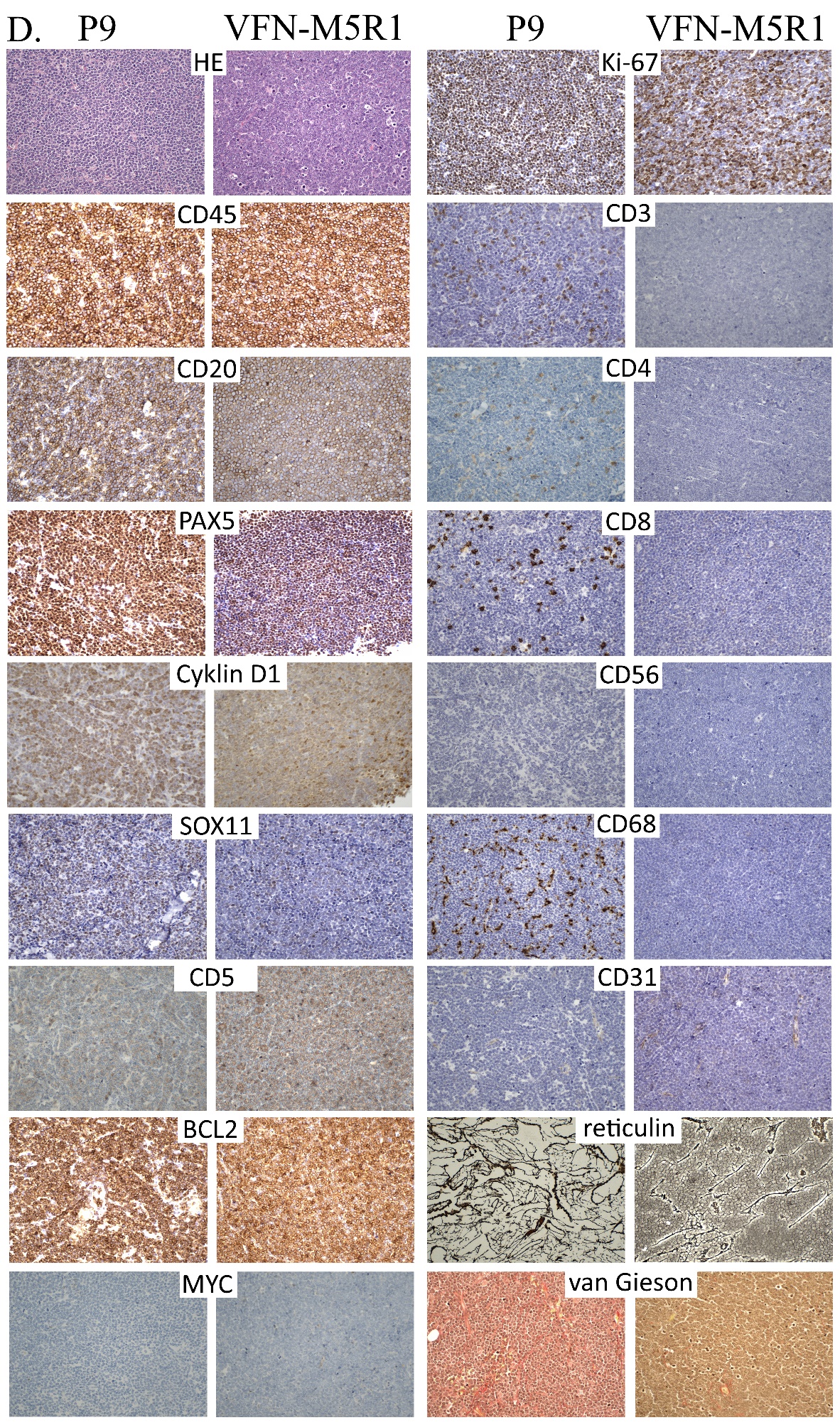
**

**Supplemental Figure 11.** Genetic and immunohistochemistry analysis of the PDX tumor VFN-M5R1 and the corresponding lymphoma biopsy (P9)

**
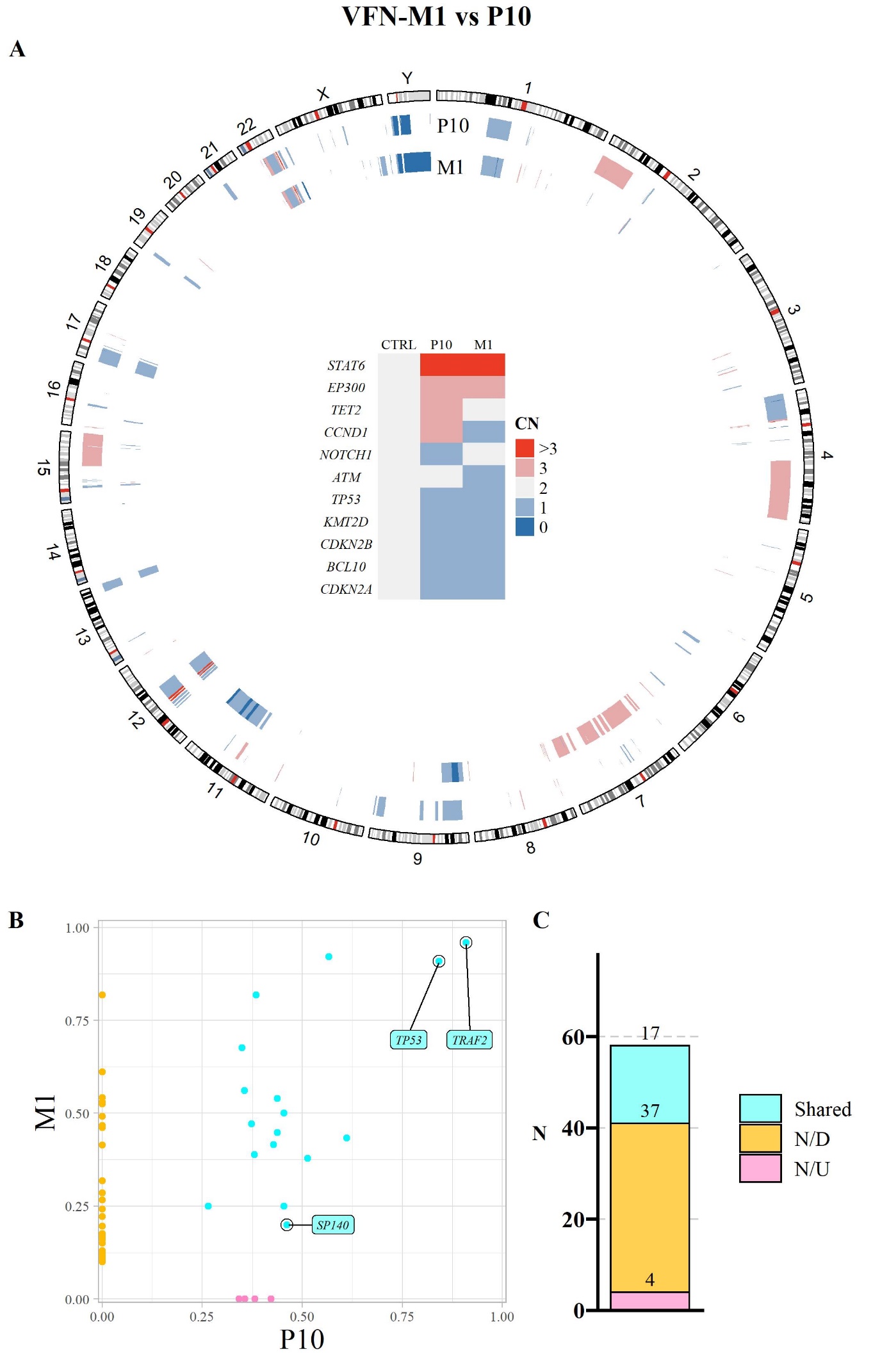
**

**
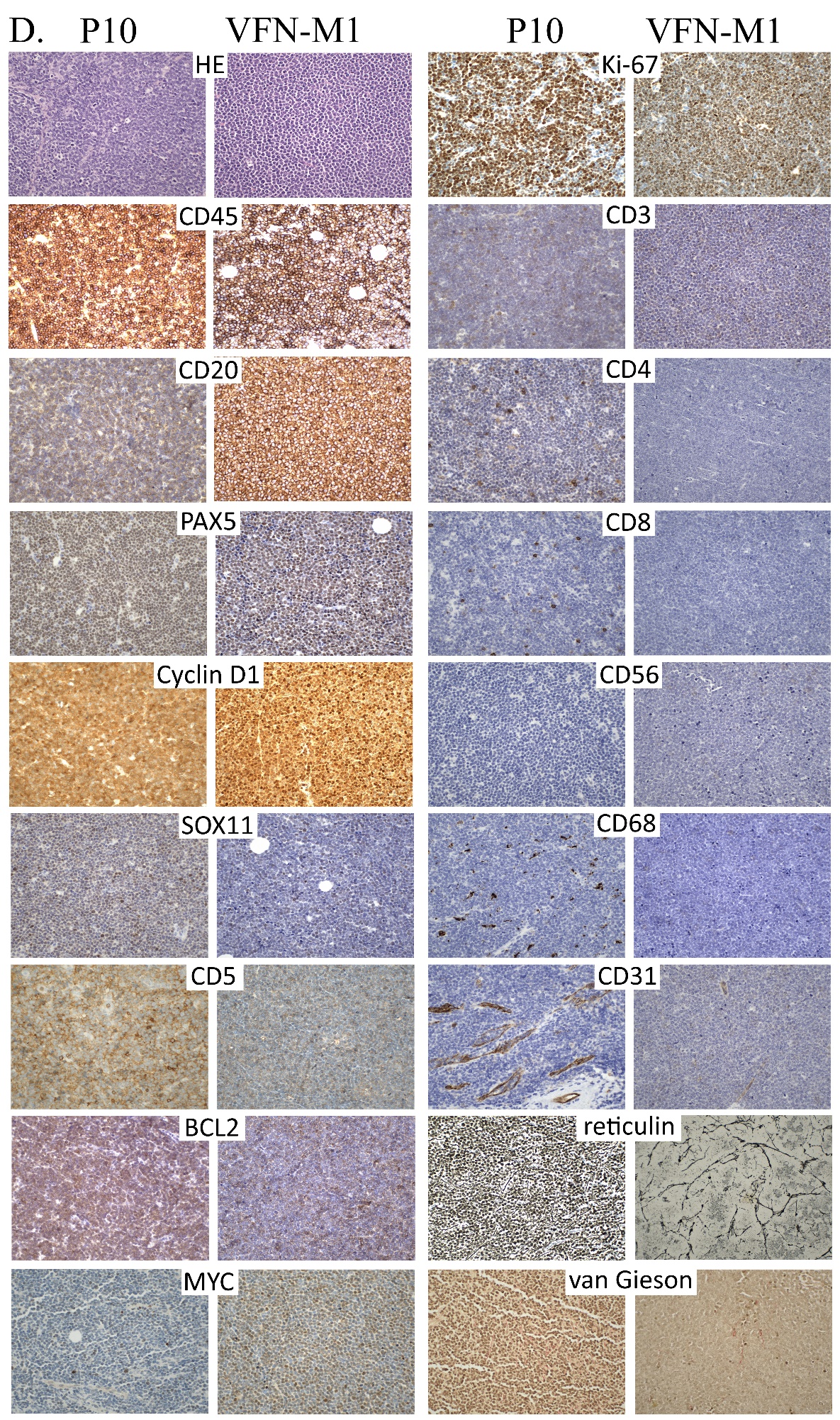
**

**Supplemental Figure 12.** Genetic and immunohistochemistry analysis of the PDX tumor VFN-M1 and the corresponding lymphoma biopsy (P10)

**
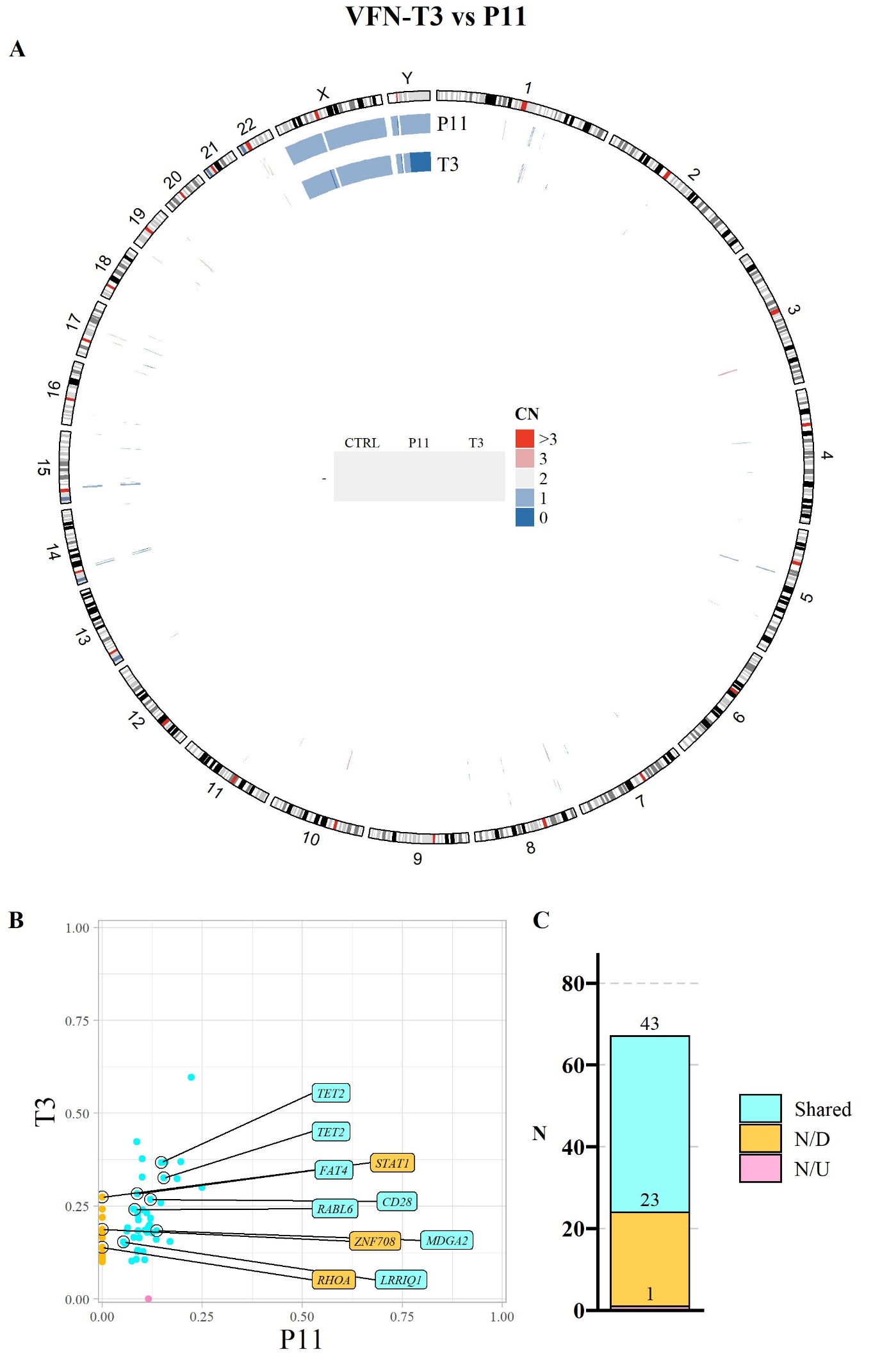
**

**
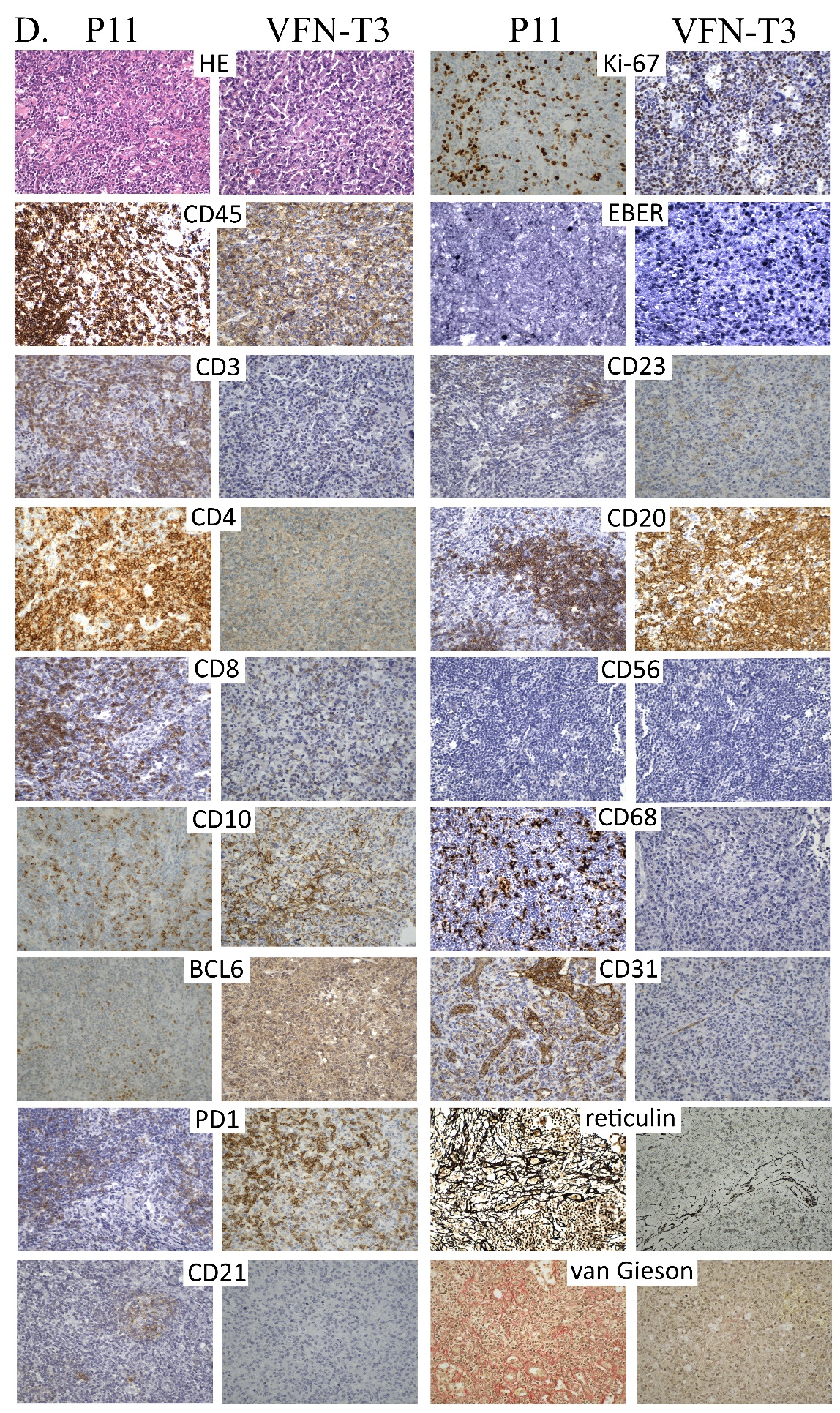
**

**Supplemental Figure 13.** Genetic and immunohistochemistry analysis of the PDX tumor VFN-T3 and the corresponding lymphoma biopsy (P11)

**
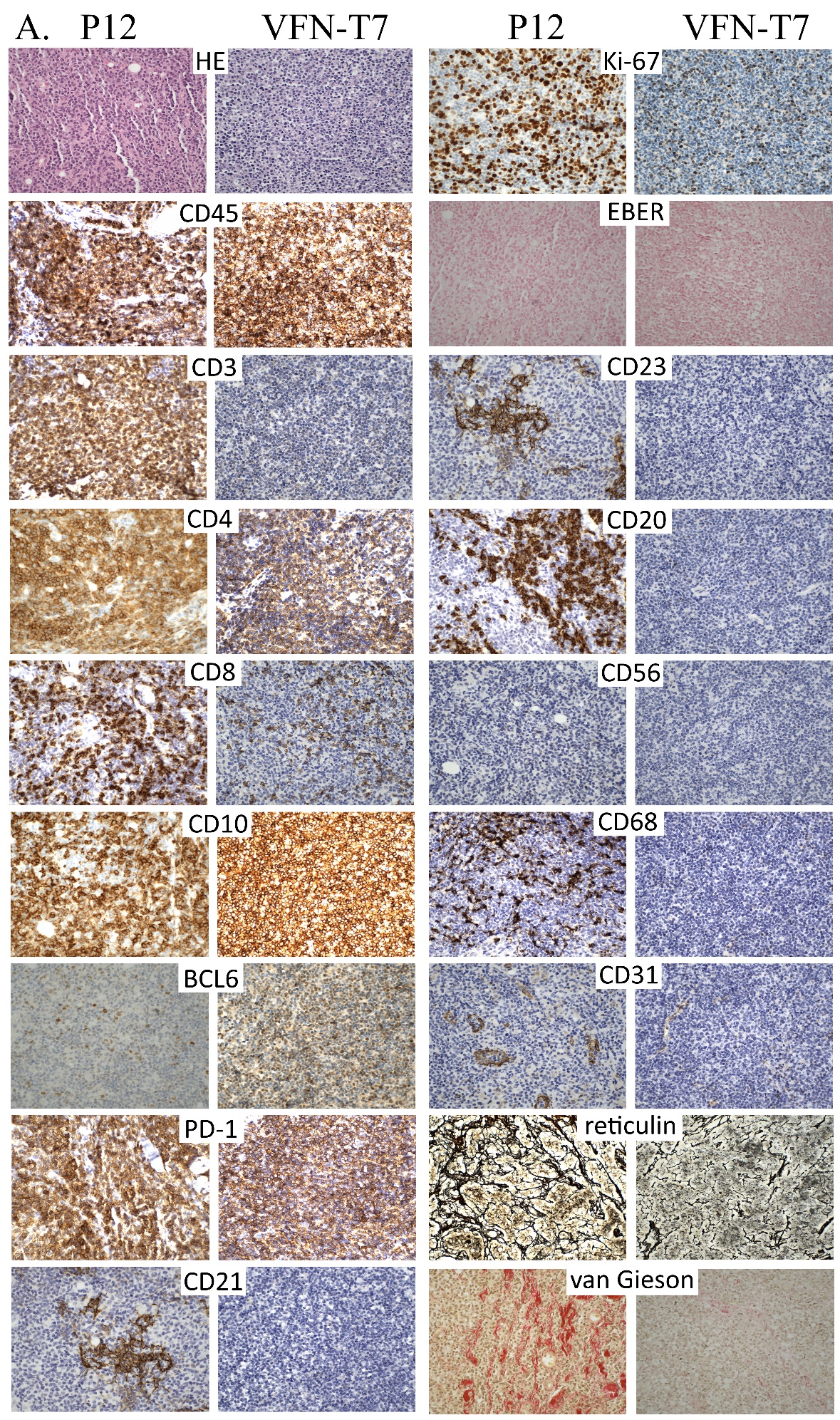
**

**Supplemental Figure 14.** Genetic and immunohistochemistry analysis of the PDX tumor VFN-T7 and the corresponding lymphoma biopsy (P12)

**
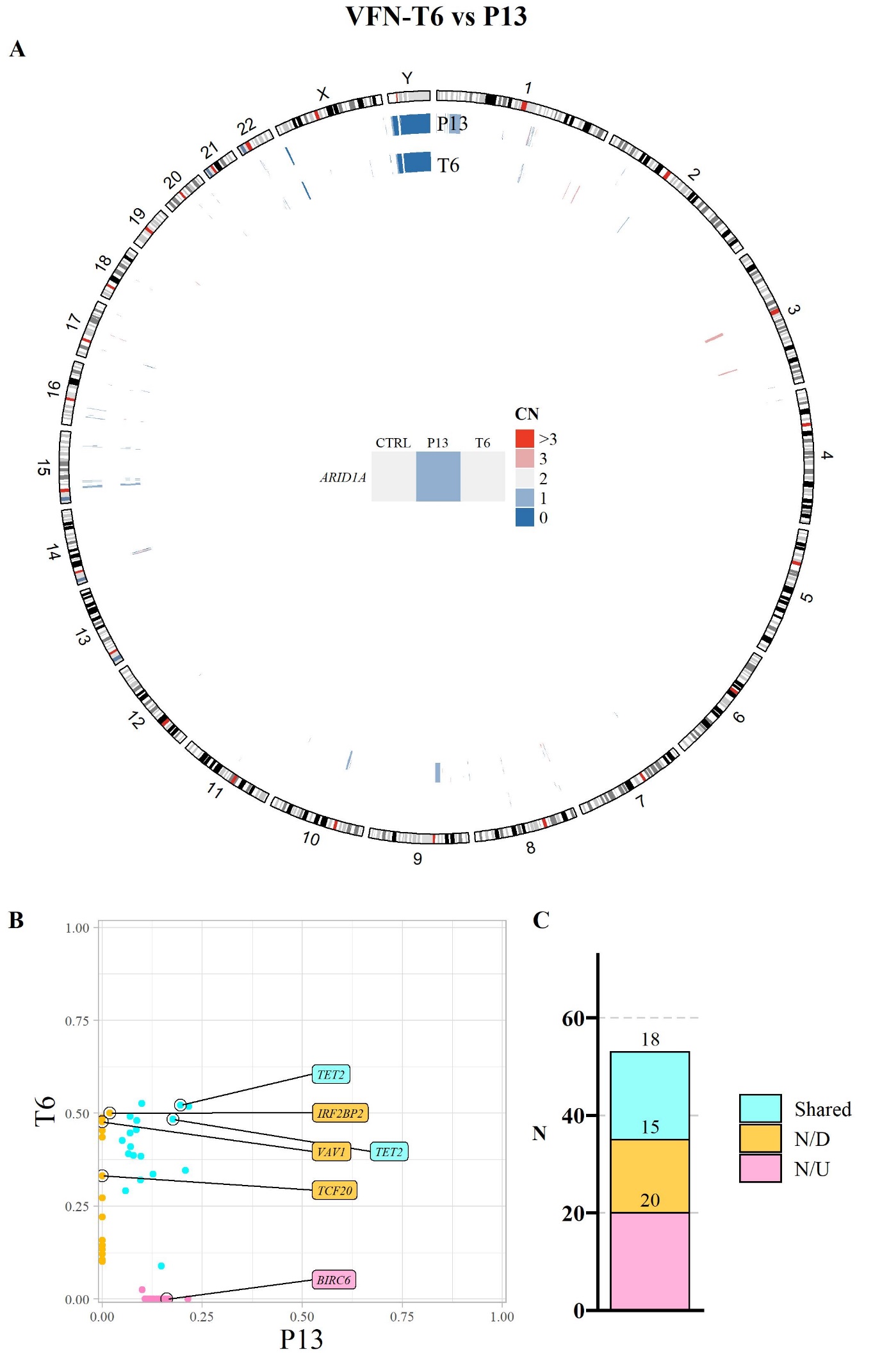
**

**
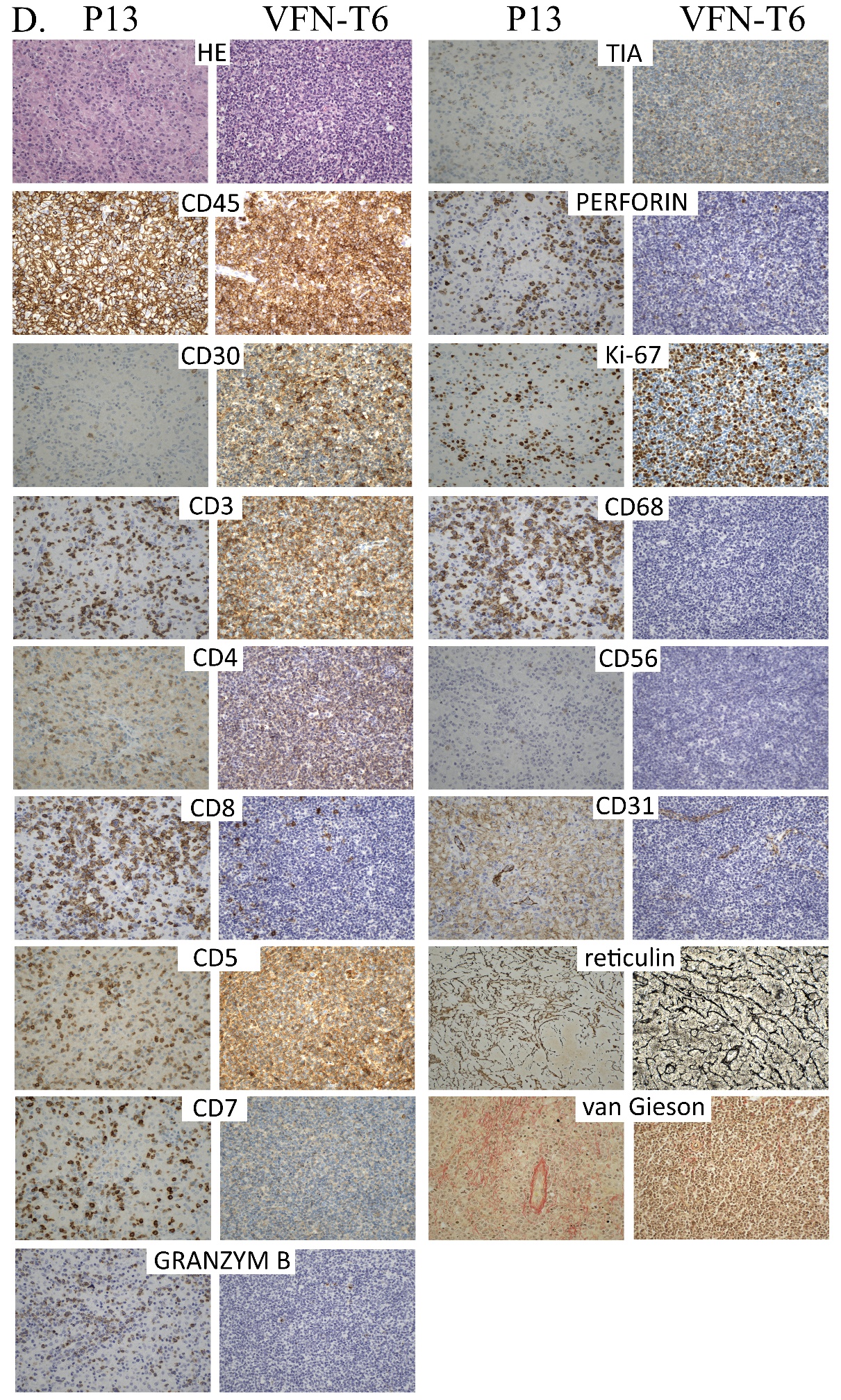
**

**Supplemental Figure 15.** Genetic and immunohistochemistry analysis of the PDX tumor VFN-T6 and the corresponding lymphoma biopsy (P13)


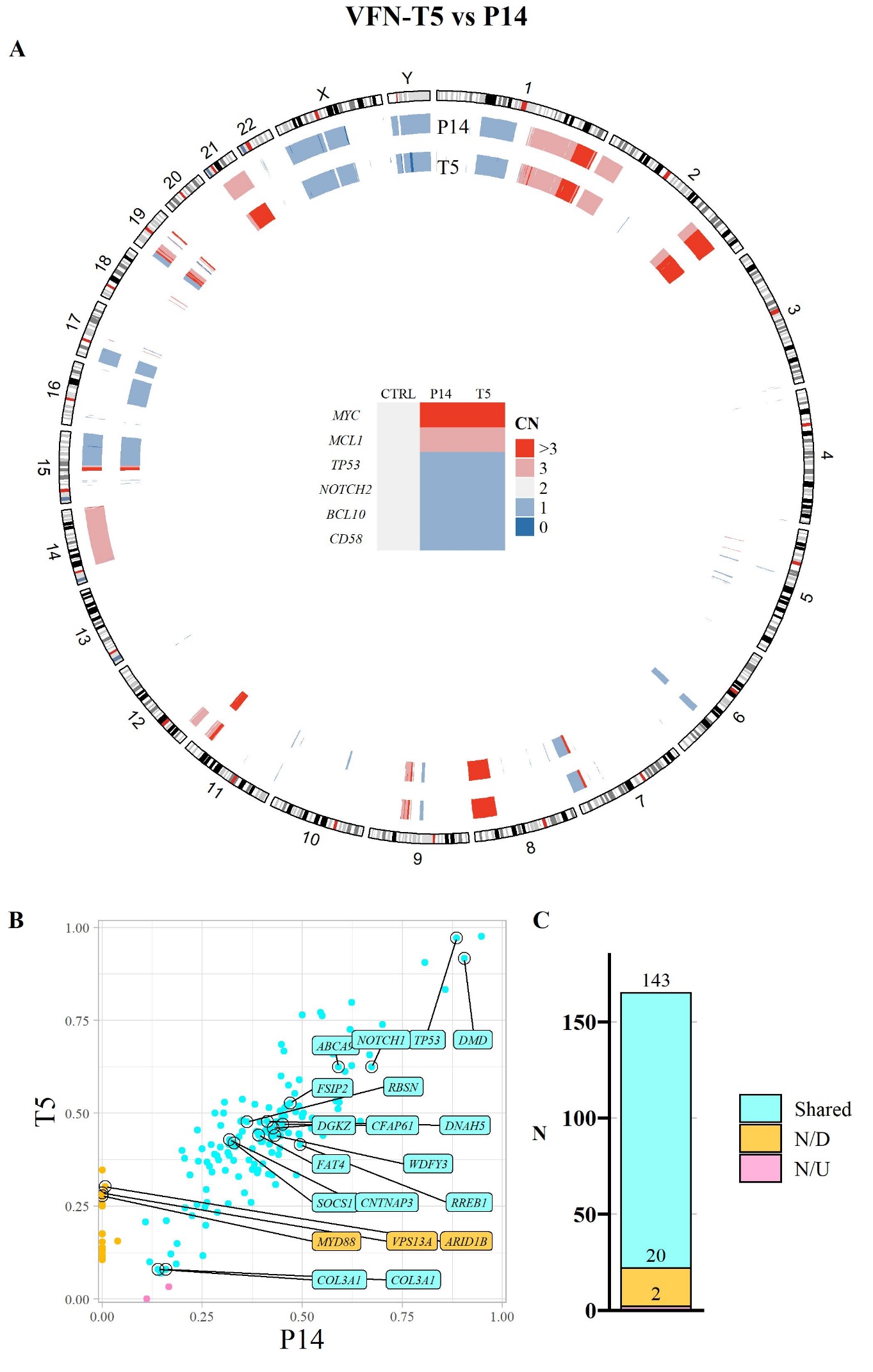


**
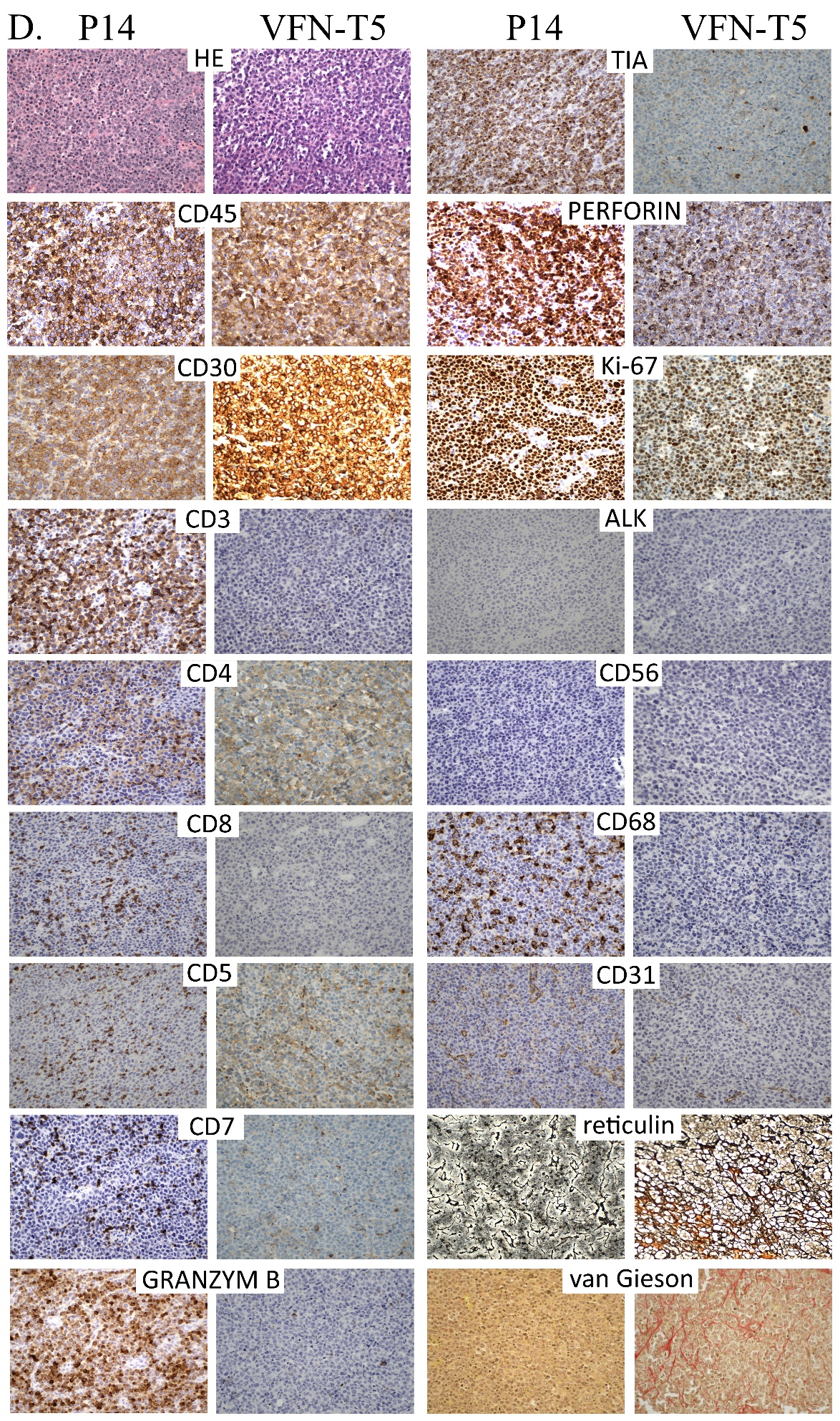
**

**Supplemental Figure 16.** Genetic and immunohistochemistry analysis of the PDX tumor VFN-T5 and the corresponding lymphoma biopsy (P14)

**
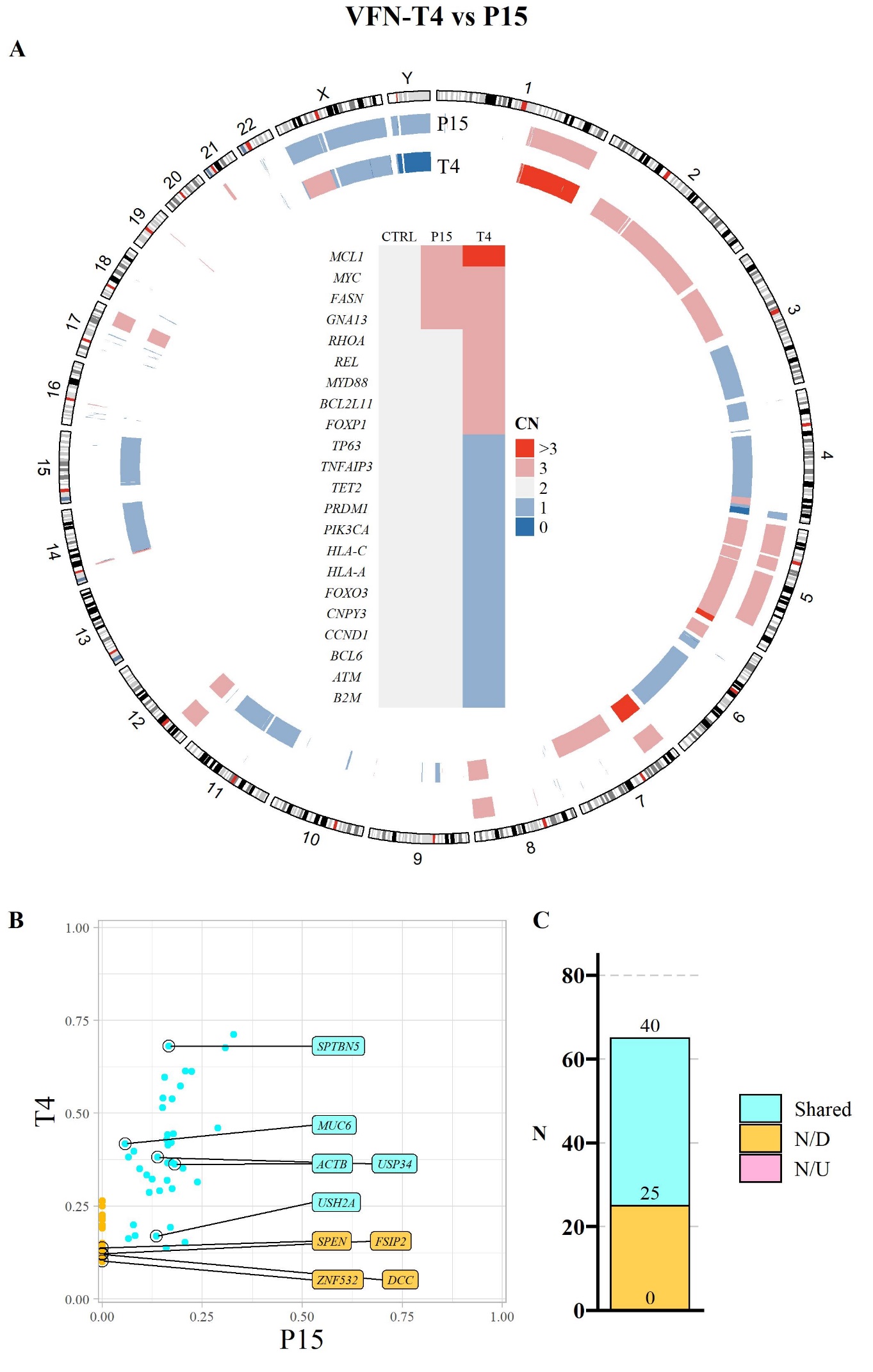
**

**
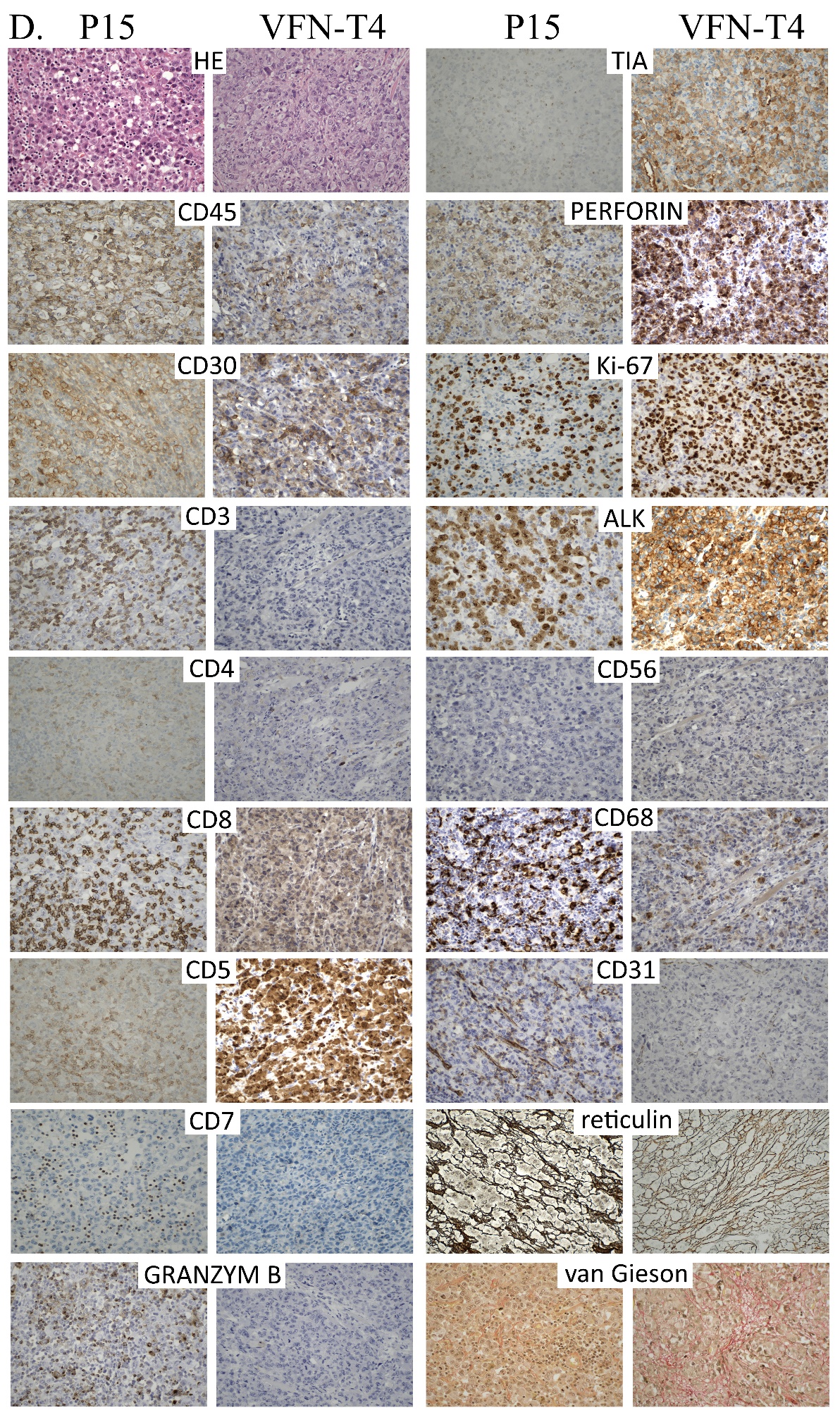
**

**Supplemental Figure 17.** Genetic and immunohistochemistry analysis of the PDX tumor VFN-T4 and the corresponding lymphoma biopsy (P15)

**Legend for Supplemental Figures 4-17:** A. Circos plots showing the copy number variation (CNV) as calculated by CNVkit for the patient’s sample (outer track) and the PDX model derived from the patient’s sample (inner track). Inferred segmental changes are marked in shades of blue (less than 2) and red (3 and more). Graphical table at the center is showing CNV in genes of special interest filtered out according to pre-established gene list for patient’s control and tumor DNA and for PDX model. **B.** Scatter plot showing the allele frequency of shared, newly detected (N/D), and newly undetected (N/U) variants in PDX model sample compared to the sample from which it was derived. Labels show variants found in genes of special interest for particular diagnosis, described in the methods. **C.** Stacked bar plots showing numbers of common, gained, and lost variants in the patient’s and the PDX’s sample. P - patient’s sample, CN - copy number, CTRL - germline control DNA from patient, N - number. D. Representative photos of immunohistochemistry analysis of primary lymphoma sample (P1) and the respective PDX model (VFN-D3); E. Evaluation of immunohistochemistry.


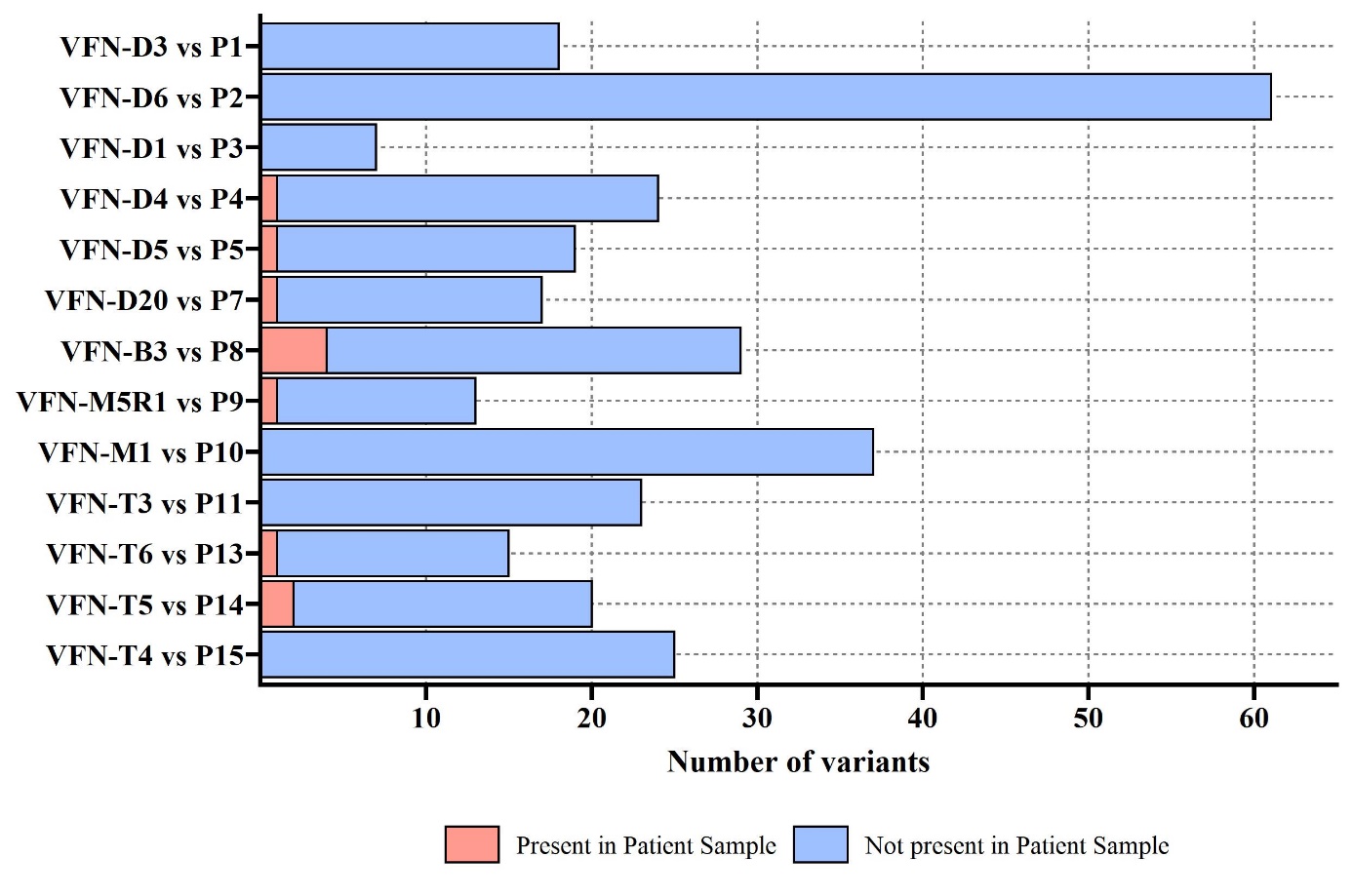


**Supplemental Figure 18.** Majority of N/D mutations were not detected in the respective patient lymphoma samples.

**Legend:** X axis: number of N/D mutations in PDX models; Y axis: N/D mutations detected in the PDX models compared to the respective patient lymphoma sample (P1-P15); blue- N/D mutations that were not detected in the respective patient primary lymphoma samples (i.e., allele frequency 0); red- N/D mutations that were detected in the respective patient primary lymphoma samples, but the allele frequency was below the specified threshold (i.e., < 10%).

**
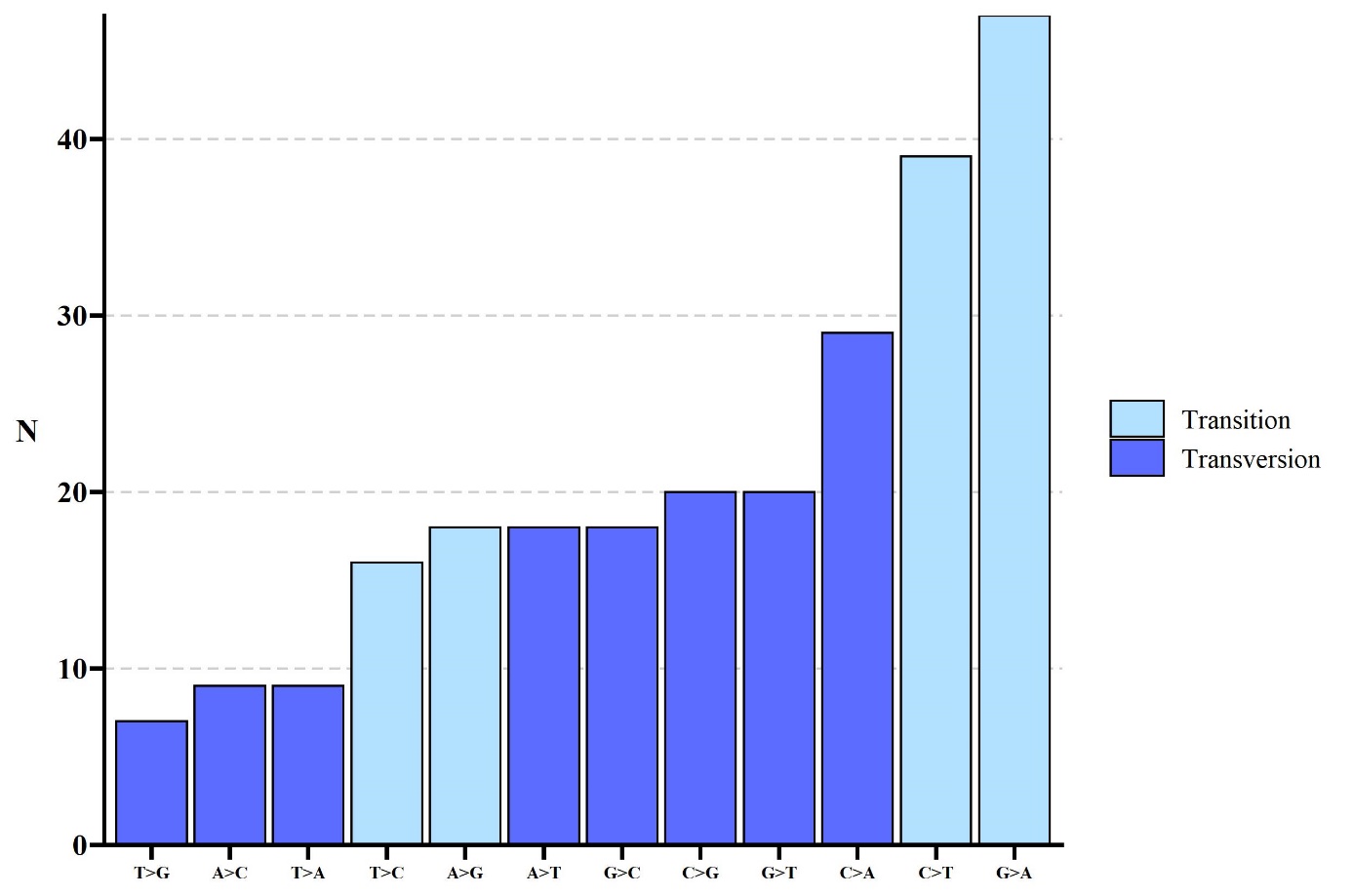
Supplemental Figure 19.** Analysis of nucleotide substitution in N/D mutations suggests contribution of somatic hypermutation process in generation of *de novo* mutations in PDX models

**Legend: A: adenine; C: cytosine; G: guanine;** T: thymine; N: number of N/D mutations in all PDX models; > stands for “substituted for”
